# A mobile healthy lifestyle intervention to promote mental health in adolescence: a mixed-methods evaluation

**DOI:** 10.1186/s12889-023-17260-9

**Published:** 2024-01-02

**Authors:** Carmen Peuters, Laura Maenhout, Greet Cardon, Annick De Paepe, Ann DeSmet, Emelien Lauwerier, Kenji Leta, Geert Crombez

**Affiliations:** 1 Department of Experimental-Clinical and Health Psychology, Ghent University, Henri Dunantlaan 2, 9000 Ghent, Belgium; 2 Department of Movement and Sports Sciences, Ghent University, Ghent, Belgium; 3 Faculty of Psychology, Educational Sciences and Speech Therapy, Université Libre de Bruxelles, Bruxelles, Belgium; 4 Department of Communication Studies, University of Antwerp, Antwerp, Belgium; 5 Department of Public Health and Primary Care, Ghent University, Ghent, Belgium

**Keywords:** Mobile health applications, Adolescents, Mental health promotion, Healthy lifestyles, Digital behavior change interventions, Participatory development, Narrative persuasion, Conversational agents, Self-regulation techniques, Universal prevention

## Abstract

**Background:**

A healthy lifestyle may improve mental health. It is yet not known whether and how a mobile intervention can be of help in achieving this in adolescents. This study investigated the effectiveness and perceived underlying mechanisms of the mobile health (mHealth) intervention #LIFEGOALS to promote healthy lifestyles and mental health. #LIFEGOALS is an evidence-based app with activity tracker, including self-regulation techniques, gamification elements, a support chatbot, and health narrative videos.

**Methods:**

A quasi-randomized controlled trial (*N* = 279) with 12-week intervention period and process evaluation interviews (*n* = 13) took place during the COVID-19 pandemic. Adolescents (12-15y) from the general population were allocated at school-level to the intervention (*n* = 184) or to a no-intervention group (*n* = 95). Health-related quality of life (HRQoL), psychological well-being, mood, self-perception, peer support, resilience, depressed feelings, sleep quality and breakfast frequency were assessed via a web-based survey; physical activity, sedentary time, and sleep routine via Axivity accelerometers. Multilevel generalized linear models were fitted to investigate intervention effects and moderation by pandemic-related measures. Interviews were coded using thematic analysis.

**Results:**

Non-usage attrition was high: 18% of the participants in the intervention group never used the app. An additional 30% stopped usage by the second week. Beneficial intervention effects were found for physical activity (*χ*^2^_1_ = 4.36, *P* = .04), sedentary behavior (*χ*^2^_1_ = 6.44, *P* = .01), sleep quality (*χ*^2^_1_ = 6.11, *P* = .01), and mood (*χ*^2^_1_ = 2.30, *P* = .02). However, effects on activity-related behavior were only present for adolescents having normal sports access, and effects on mood only for adolescents with full in-school education. HRQoL (*χ*^2^_2_ = 14.72, *P* < .001), mood (*χ*^2^_1_ = 6.03, *P* = .01), and peer support (*χ*^2^_1_ = 13.69, *P* < .001) worsened in adolescents with pandemic-induced remote-education. Interviewees reported that the reward system, self-regulation guidance, and increased health awareness had contributed to their behavior change. They also pointed to the importance of social factors, quality of technology and autonomy for mHealth effectiveness.

**Conclusions:**

#LIFEGOALS showed mixed results on health behaviors and mental health. The findings highlight the role of contextual factors for mHealth promotion in adolescence, and provide suggestions to optimize support by a chatbot and narrative episodes.

**Trial registration:**

ClinicalTrials.gov [NCT04719858], registered on 22/01/2021.

**Supplementary Information:**

The online version contains supplementary material available at 10.1186/s12889-023-17260-9.

## Background

The prevalence of mental health problems among youth is high [1, 2] and costly [3, 4]; chief amongst these are depression and anxiety. Because half of all mental disorders emerge around the age of 14, early adolescence provides a window of opportunity for mental health promotion [5, 6]. One way to promote mental health is to teach adolescents socio-emotional and cognitive competences that help them cope with stress and strengthen their resilience [7, 8]. Another way is to encourage adolescents to adopt and maintain a healthy lifestyle [9, 10].

Lifestyle behaviors that may protect mental health include, amongst others, regular physical activity [11, 12], reduced sedentary time [13, 14], adequate sleep [15, 16], and a good quality breakfast as part of a healthy diet [17]. Providing adolescents with the necessary skills to adopt a healthy lifestyle may empower them to have more control over their physical and mental health [18]. A preventive intervention focusing on healthy lifestyles may furthermore be less stigmatizing for students struggling with mental health issues compared to interventions that directly focus on mental health and target those at risk for mental health problems [19].

Interventions to promote health behavior are increasingly delivered by mobile devices [20]. So-called ‘mobile health’ or ‘*mHealth’* is especially suitable for adolescents considering the integration of smartphones in their daily life [21]. There is a growing number of mHealth interventions targeting the promotion of one or more healthy lifestyle behaviors in adolescents. Despite their high feasibility and acceptability, only about half of the mHealth intervention studies show an effect on health behavior [22, 23].

Several principles have been proposed for creating effective mobile health behavior change interventions. First, a human-centered design approach comprising an iterative, interdisciplinary, and collaborative development process, is proposed to address the needs and preferences of end-users [24, 25]. A good fit with the end-user and context may ensure that the intervention will be relevant for the target group and improve its adoption [10, 24]. Second, behavior change theories for identifying and targeting relevant environmental and personal determinants of behavior change should form the basis of the intervention [26, 27]. In particular, the provision of self-monitoring and feedback, the inclusion of reminders and notifications, and the use of rewards and gamification, have been put forward as promising strategies in mHealth for engaging youth towards healthier behavior [28]. Third, an mHealth technology needs to be persuasive to ensure attractiveness and engagement, and to be convincing to change behavior [24]. One key persuasion strategy is the complementarity of direct route information processing (i.e., through explicit and reflective elaboration of the health information) with indirect routes (i.e., through implicit and automatic processing of information) (see Persuasive Systems Design model [29]). Especially for adolescents with low motivation or ability to process the health message, it is recommended to first communicate via an indirect route such as interweaving the health message in storytelling [30]. The hypothesis for narrative communication is that when the audience becomes absorbed in an engaging story and can identify with a main character, they will change their health attitudes according to the storyline [31]. Fourth, it is important to consider a social support feature in a mobile behavior change intervention [28, 32]. A chatbot providing human-like interaction has potential as interactive feature to facilitate the receipt of social support and increase user engagement [33]. Previous studies show that a chatbot was received positively by adolescents as an innovative way to answer health questions [34] and that it might be effective for improving healthy lifestyles [35] and mental health [36].

### Goal of this study

Only few mobile interventions for health behavior change have been developed to promote mental health, and they generally focus on mindfulness meditation or emotional regulation rather than on healthy lifestyles [37]. Moreover, mHealth interventions often target specific groups, and few are universal [38]. A health promotion approach targeting the general population, including non-clinical groups, can prevent problems and related costs at later age. Therefore, we developed the mHealth intervention #LIFEGOALS, a theory-based health promotion app integrating evidence-based techniques with engagement-enhancing features to promote mental health by supporting the adoption of a healthy lifestyle. This study reports the effects of #LIFEGOALS on mental health and health behaviors, and the insights from users on the working processes of the different intervention components.

## Methods

### Study design

**Fig. 1:**
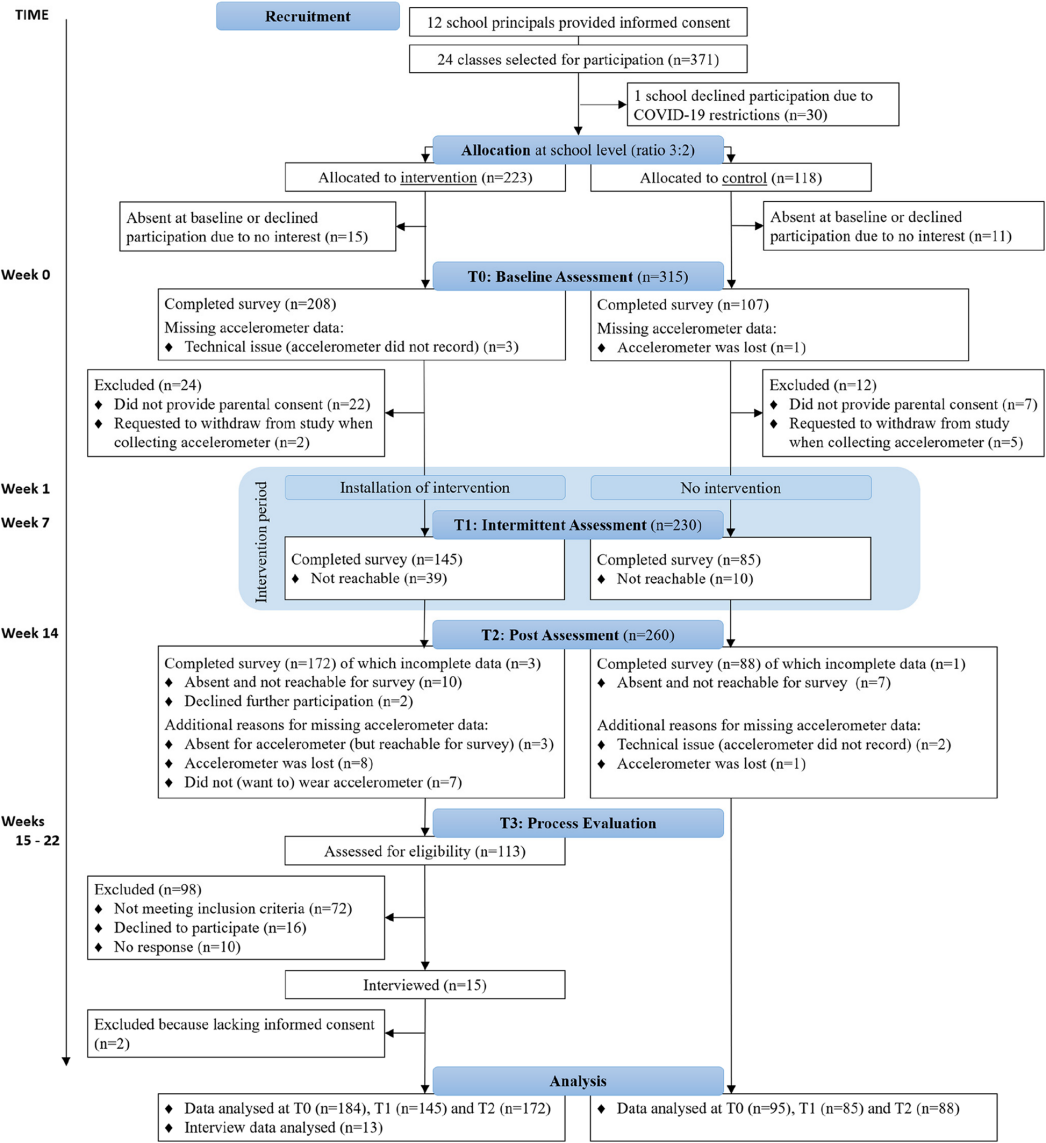
Study design and participant flow

This study used a mixed-methods two-armed cluster-controlled trial with process evaluation (Fig. 1). Participants were assigned to either the intervention or control group in a 3:2 ratio to allow for more data on usage of the intervention. Participants in the intervention group received the #LIFEGOALS intervention over a period of 12 weeks. Participants in the control group received no intervention. Mental health and behavioral outcomes were collected at baseline (T0) and post-intervention (T2). Health-related quality of life (HRQoL), the primary outcome, was also collected after six weeks of intervention (T1). User perception of possible working processes were assessed via individual interviews in the eight weeks following post-intervention assessment (T3). The study was preregistered at Open Science Framework [39] and is reported according to the CONSORT-EHEALTH checklist v.1.6.1.

### Participants and recruitment

The #LIFEGOALS project targeted adolescents in the first three years of secondary education (aged 12 to 15 years) in Flanders (Belgium). Inclusion criteria were being proficient in Dutch. Exclusion criteria were attending special needs education or education for foreign-language speaking children. From June 2020 on, eligible schools were selected from a public database via a combination of random and convenience sampling. Schools received information about the study via e-mail, followed up by a phone call to discuss potential participation. To keep the cluster effect as small as possible, we aimed to recruit a minimum of nine schools. At the end of September 2020, 12 schools from 5 provinces in Flanders provided consent, and were invited to select one or more classes of between 20 and 40 students. Due to restricted COVID-19-related measures, one school had to withdraw participation before data collection had started.

The process evaluation was undertaken in a subsample of the intervention group. Only participants from the second and third batch of data collection (i.e., data collection for the trial started at three different moments) were included, because during the first batch there were some technical problems with the intervention which were corrected by the start of the second batch. Eligible were those participants who engaged with the app for 20 min or more over the 12-week intervention period, as this was considered to reflect sufficient duration for purposeful usage. Participants were selected by means of stratified randomization to maximize variation in school, study year and gender. Invited participants were offered an online voucher of €20 as incentive, and recruitment continued until data saturation occurred.

### Allocation

Stratified allocation to the intervention or control group occurred at school level. Adhering to the allocation ratio of 3:2, of schools with similar characteristics (i.e., same education type and school grade), the school with the highest number of selected students was assigned to the intervention group and the other school to the control group.

### Data collection and procedure

#### Quasi-RCT

Data were collected between October 2020 and May 2021 (during the second and third wave of the COVID-19 pandemic in Flanders, Belgium). At baseline (T0), the study was explained to the participants, after which accelerometers were distributed with the instructions to wear the device day and night for the following seven days. Participants then filled out a web-based survey assessing sociodemographic information, mental health, and health behavior. Upon completion they received a first incentive (power bank for the value of €3). One week later, the accelerometers were collected. At that point, the intervention group received the #LIFEGOALS app together with a Fitbit Charge 2 or 3 (for self-monitoring as part of the intervention, not for measurement). Researchers helped install the #LIFEGOALS app on the participants’ smartphone, and helped connect the Fitbit to the app. A short presentation was given to explain how to use the app. No time was provided to explore the app. The control group was not provided an intervention, nor a Fitbit. Seven weeks after baseline, participants completed a brief web-based survey assessing HRQoL (T1). Due to the pandemic-related measures, not all students were allowed full-time in-school education, which is why some classes were instructed to complete the survey at home during remote education. Seven weeks later, at post-intervention measurement (T2), participants completed a final web-based survey assessing mental health and behavioral measures, and again accelerometers were handed out (after collecting the Fitbits in the intervention group). Participants who had worn their accelerometer correctly at baseline and had completed all previous surveys, received an additional incentive (cinema voucher for the value of €10). The accelerometers were collected one week later, and if worn correctly participants received a (second) cinema voucher.

#### Process evaluation interviews

Interviews were conducted online using Microsoft Teams. CP, KL, and four master thesis students trained in interview skills, followed a semi-structured interview guide (see Table 1). All interviewers had been involved in the data collection and were familiar with the app. Support and guidance were provided by EL, who has extensive experience in conducting qualitative research interviews. Interviews lasted between 15 and 45 min and were audio and video recorded.

**Table 1:** Topics and guiding questions of the interview

Topic	Interview guide question
Spontaneous impression	– What comes to your mind when you think of the #LIFEGOALS app?
Feasibility	– How was it to use the app? – Why was it (not) easy to use? – What did you (not) enjoy? – What did you think about the looks of the app?
Mechanisms of action	– With what has the app helped you? – How/in what way did the app help you with that? / Why didn’t the app help with anything?
Used as intended?	– Can you tell us some more about [component X]?^a^ – How have you used [component X]?^a^
Context and usage	– When and where have you used the app? – What has (de)motivated you to use the app? – How did others (peers, family, teacher) feel about the app? What influence did this have on you?

^a^ Asked for each component of the app separately

### #LIFEGOALS intervention

#LIFEGOALS is a multi-component mHealth intervention and was introduced as an entertaining health app to improve lifestyle. The intervention targets four lifestyle behaviors: increasing physical activity, reducing sedentary behavior, improving adequate sleep, and daily taking breakfast. The intervention included: (a) self-regulation features among which goal-setting, action and coping planning, and self-monitoring (using a Fitbit) to help bridge the intention-behavior gap; (b) a health narrative in the format of brief (3 to 6 min) weekly video episodes with an entertaining storyline evolving implicitly around lifestyle behaviors for mental health (videos available on YouTube [40]); (c) an automated chatbot for individual support by providing information and sending supporting messages; and (d) other behavior change techniques (BCTs) (rewards, information) for persuasion and raising knowledge. Figure 2 shows the home screen of the #LIFEGOALS app. Although the intervention was not introduced as a tool to improve mental health, information on the benefits of lifestyle behaviors for mental health could be found in the information section and chatbot of the app. The use of the intervention was stimulated by installing a roll-up banner with motivational visuals at a visible place in the classes. Users were free in when, where, what components and how often they used the intervention. No internet connection was required to use the app (only to load in the Fitbit data for self-monitoring and to watch the narrative video episodes).

**Fig. 2:**
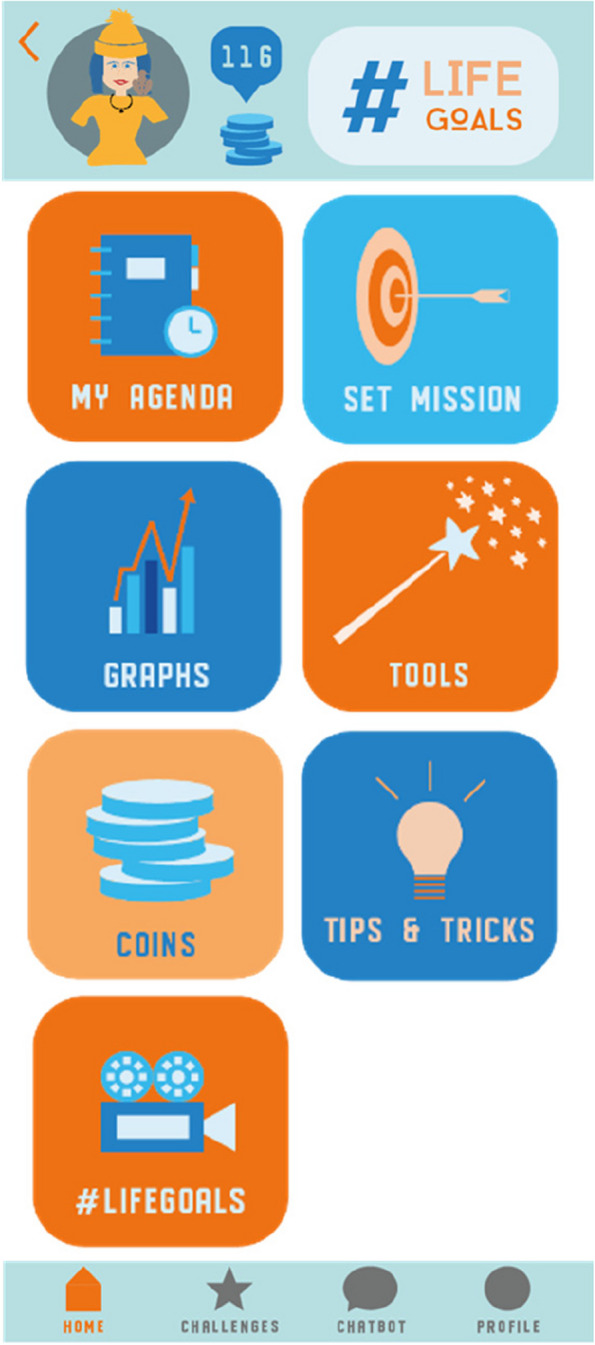
Home screen of the #LIFEGOALS app

The intervention design was informed by the Health Action Process Approach (HAPA) model [41] which includes self-regulation techniques to move from an intention to change behavior to actual behavior change, by the Elaboration Likelihood Model (ELM) [30] which theorizes that information processing may either occur via a direct or via an indirect route depending on the motivation and ability of the individual to elaborate on the message, and by the Persuasive Systems Design model (PSD)[29] which proposes several key principles for a persuasive design. Stakeholders and end-users were actively involved throughout the entire development process. More detailed information on the content, theoretical rationale, and development process of the #LIFEGOALS intervention is publicly available on Open Science Framework [42].

During the first weeks of intervention (affecting participants of the first batch of data collection), technical bugs were detected and resolved. These were related to the agenda (e.g., the impossibility to link the app with the smartphone’s agenda during installation, problems with compatibilization with a smartphone’s agenda in AM/PM instead of 24 h, or the impossibility to delete a planned activity from the agenda), certain crashes (e.g., when using the avatar), structural mistakes (e.g., a missing ‘own idea?’-button or check marks that were wrongly marked by default), or notifications by the chatbot (the failure of a badge to appear when receiving a new message). Participants from the first batch of data collection were sent an email with the instructions to update the #LIFEGOALS app with the new version.

### Measures

#### Demographic variables

In Flanders (Belgium), secondary school students in the first two years follow either the A- or the B-track depending on their learning abilities (students with learning disabilities follow the B-track). From the 3rd year on, students from the A-track can choose between ‘general’, ‘technical’, ‘art’ or ‘vocational’ tracks, and students from the B-track follow the ‘vocational’ track. To obtain a coding system that is equal for all school grades, *education type* was categorized into ‘general or technical track’ (A-track, general, technical and art education) and ‘vocational track’ (B-track and vocational education). *Family affluence* was assessed with the revised Family Affluence Scale (FAS) [43]. The scale consists of 6 indicators of family welfare. However, one item (“How many times did you travel abroad for holiday/vacation last year?”) was omitted due to the pandemic-related restrictions on travelling outside of Belgium. This resulted in a FAS sum score ranging from 0 (low affluence) to 10 (high affluence). Flemish FAS-data from the 2017/18 Health Behavior in School-Aged Children (HBSC) study [44] were used to determine the cut points for this reduced FAS sum-score to create low (0–6), middle (7–8) and high (9–10) affluence groups, responding to respectively 21.1%, 56.6% and 22.2% of the Flemish youth population.

#### Mental health

*Health-related quality of life (HRQoL)* was the primary outcome and was measured with the KIDSCREEN-10 [45]. To get a more in-depth picture of participants’ mental health, also the subscales *psychological well-being* (i.e., positive emotions, satisfaction with life, and feeling emotionally balanced) and *social support & peers* (i.e., quality of interaction with friends and peers, and perceived support; further referred to as peer support) from the KIDSCREEN-27 [46], and *moods & emotions* (i.e., depressive moods and emotions and stressful feelings; further referred to as moods) and *self-perception* (i.e., self-confidence, self-satisfaction and body image) from the KIDSCREEN-52 [47], were assessed. The KIDSCREEN-10 and the used subscales have shown good reliability and validity [48, 49]. For each construct, the scores on the items were transformed into t-values (mean 50, SD 10) using the European norm data for adolescents [50] and averaged, with higher scores indicating better well-being.

*Resilience* was measured with the Brief Resilience Scale (BRS) [51]. We added a timeframe and adapted the wording of the Dutch version of the BRS [52] to make the items adequate for young adolescents. This was discussed in team, and the adapted questionnaire was tested on comprehensibility with four adolescents. A higher mean score indicates greater capacity to bounce back after hard times.

*Depressed feelings* was measured using the Custom Short Form of the Dutch version of the Patient-Reported Outcomes Measurement Information System Pediatric Bank Depressive Symptoms v2.0 (PROMIS-PedDepSx) [53], showing good psychometric properties [54]. The four items were transformed into t-values (mean 50, SD 10) using the health measures scoring service provided by PROMIS. Scores were averaged with a higher score indicating more depressed feelings.

#### Lifestyle behaviors

Physical (in)activity and sleep were measured using the 3-axis accelerometer Axivity AX3. Axivities were worn on the non-dominant hand in a black wristband without display. These trackers are not to be confused with the Fitbit that was provided to the intervention group, where measurements are visible to the user and that were used as self-monitoring tool in the intervention. Accelerometers were configured to record raw acceleration data at a frequency of 100 Hz with a range of ± 8* g* using Open Movement GUI (OMGUI, V1.0.0.43). Raw accelerometer data (.cwa) were downloaded using the same software and were processed in R v4.0.5 using the GGIR package v2.3–0 [55]. Non-wear time was calculated based on the algorithm of Van Hees et al. [56]. A valid day was set at a minimum of 10 h of wear time between 3 a.m. and 3 a.m., a valid night at a minimum of 8 h of wear time between 9 p.m. and 11 a.m. Participants with a minimum of 2 valid weekdays/weeknights and 1 valid weekend day/night, were included in the analyses concerning accelerometry activity/sleep data.

The Euclidean Norm Minus One (ENMO) was used as parameter for *physical activity*. The ENMO metric is a measure of the total volume of physical activity and is expressed in milligravity-based acceleration units (*mg*). ENMO was calculated using step 1–2 of the GGIR package v2.3–0 in R [56], applying imputation for missing data such as monitor non-wear. The average ENMO per awake minute was calculated over the valid days per participant per assessment point. Phillips cut points [57] were used to classify minutes into light, moderate or vigorous activity intensity levels. All minutes with an ENMO of less than 250* mg* (Phillips cut point) and not classified as sleep, were considered *sedentary time*. The total amount of sedentary minutes was calculated over the valid days per participant per assessment point.

Weekday-to-weekend sleep time difference was used to measure participants’ *sleep routine*. A smaller sleep time difference is associated with better mental and (to a lesser extent) physical health [58]. The estimation of stationary sleep-segments was calculated using the OMGUI software (implementing the algorithm by Borazio et al. [59]), upon which minutes of detected sleep between 9 p.m. and 11 a.m. were summed to become sleep time per night. The following corrections were made: a) observations were detected as sleep if the interruption of non-sleeping bouts was less than 30 s, and b) observations were not considered sleep if a sleeping-bout was less than 30 min. Non-wear within the 9 p.m. – 11 a.m. sleep window was considered sleep if it lied between the average sleep–wake hours of Flemish Belgian young adolescents (i.e., 9:45 p.m.—6:49 a.m. on weeknights, or 11:14 p.m.—9:37 a.m. on weekend nights [60]). Sleep routine was calculated by subtracting the average minutes of sleep during the week (from Sunday evening to Monday morning) from the average minutes of sleep during the weekend (from Friday evening until Sunday morning).

Sleep quality and breakfast frequency were assessed in the web-based survey. For *sleep quality*, we selected items from the HBSC 2017/18 study [44] and the Adolescent Sleep Wake Scale [61] to assess four core elements determining sleep quality: experience of sleep quality, sleep latency, sleep interruption, and daytime sleepiness. The items referred to the past week and were scored on a 5-point Likert scale (totally disagree – totally agree). A higher mean score indicates better perceived sleep quality. The *frequency of taking breakfast* was measured with two items from the HBSC 2017/18 study [44], assessing how often the participant usually takes breakfast on school days and weekends.

#### Log data

*In-app duration* was operationalized by summing all time in between opening the app, and leaving the app by either closing it, switching to another app or phone content, or the phone entering into screen-lock. In-app duration over the intervention period spanned the day of installation until exactly twelve weeks later.

#### Pandemic-related measures

During the second wave of the COVID-19 pandemic in Belgium (at the start of data collection), the Belgian government implemented measures to limit spread of infections. The measures with the greatest impact on 12- to 15-year-olds concerned restrictions for in-school education and sports (see Additional file 1). Two variables were created to reflect the measures in force regarding education (yes/no ≥ 50% remote education) and sports (yes/no restrictions to perform sports activities).

### Data analyses

#### Quantitative analyses (quasi-RCT)

##### Sample size

Sample size calculation was performed for a design with imbalanced allocation ratio of 3:2, to detect a difference of 5 points in the mean score on the KIDSCREEN-10 [62], assuming a power of 80%, and a level of evidence of 5% using a random intercept 2-level (school and student) nested model with a variance between schools of 4.55 and a residual variability of 140 [63]. To allow for a predicted drop-out rate of 20%, the required sample size of 216 participants was raised to a total of 270, requiring 180 participants in the intervention group and 90 in the control group.

##### Statistical analyses

Analyses were performed in R version 4.0.5 with statistical significance determined at *α* = 0.05. To examine the intervention effects, the intervention group was limited to the participants with a minimum in-app duration of 2 min. This selection perfectly coincided with the group of participants who in the post-survey answered ‘yes’ (compared to ‘no’) to the question whether they had taken a look at the app. For each outcome, a multilevel generalized linear model with random subject intercept was selected based on Akaike’s Information Criteria (AIC). A generalized linear mixed model with Gamma variance function and identity link function was selected for all mental health outcomes, physical activity, and sleep quality; and a Gaussian generalized linear mixed model with identity link function was selected for effects on sedentary time, sleep routine, and breakfast frequency. Age, gender, and family affluence were included as covariates. For all the models, including school as random intercept did not significantly improve the fit of the model. Models were run to investigate change in the dependent variables from baseline (T0) to post-intervention (T2), and for HRQoL from baseline (T0) to intermittent (T1) to post-intervention (T2). Because pandemic-related measures may influence the intervention effects, it was checked whether pandemic-related education restrictions (for each outcome) and sports restrictions (for the activity-related outcomes physical activity and sedentary behavior) moderated the intervention effect. A three-way interaction between Time (T0, T1, T2), group (intervention vs. control) and pandemic-related restrictions (yes/no) was included in the multilevel generalized linear models. In case of moderation, post hoc analyses were performed to analyze the intervention effects for the specific subgroups (no restrictions vs. restrictions).

#### Qualitative analyses (process evaluation interviews)

Interviews were transcribed verbatim and coded and analyzed in NVivo 1.5.2 using thematic analysis [64, 65]. CP, EL, and a master student performed an in-depth analysis of one interview, upon which a coding scheme was created. Data were analyzed with the aim to explore the most salient intervention elements and working processes that improved health behavior or mental health (as experienced by the users), and to serve as an explanatory ground for the quantitative results. The same group of coders independently analyzed a next set of six interviews based on the pre-determined coding scheme, and new themes that emerged from the data were added. The remaining interviews (n = 6) were analyzed by two coders independently (CP and master student). In between, findings were discussed within the research team and led to subsequent adding or rewording of themes. The final coding scheme consists of subject areas, description of those areas and illustrative quotes. The findings were written out in a report that was read and revised by all authors, ensuring credibility of the reporting process.

## Results

### Quasi-RCT

#### Drop-out, adherence and demographics

The flow of participants through recruitment, allocation and data collection is presented in Fig. 1. In total, of the 315 adolescents who completed baseline assessment, 36 (11,43%) did not provide full informed consent or explicitly indicated to withdraw participation. Of the 279 participants included in the study (mean age 13.63, SD 0.96), drop-out at posttest was 6.81% for survey data and 14.34% for accelerometer data. Of the 184 participants in the intervention group, 25 (13.59%) could not install the app on their smartphone (often because parental approval was needed to install an app, or because under 13-year-olds are not permitted to use test-versions of apps on iPhone), and 9 (4.89%) only installed the app but did not use it. Of the 150 participants who used the app, 56 (37.33%) only used the app in the first week, and an additional 44 (29.33%) stopped using the app within the first half of the intervention. Only 26 users (17.33%) reported having watched three or more of the twelve episodes of the narrative. Attrition rates of this study are discussed in more detail elsewhere [66]. Median in-app duration was 19 (MAD 15) minutes. Table 2 shows for each health behavior the percentage of participants who chose to work on the behavior.

**Table 2:** Behavior focus of action plans (*N* = 150)

Behavior	n (%)	Median (IQR)^a^
Physical activity (move more)	50 (33.33)	1.5 (2)
Sleep (get enough sleep)	41 (27.33)	1 (1)
Breakfast (have daily breakfast)	36 (24.00)	1 (1)
Sedentary behavior (sit less)	25 (16.67)	1 (0)
No action plans	68 (45.33)	-

^a^Average number of action plans of the participants setting ≥ 1 plan for the specific behavior

The analyses for this paper were run on the 150 participants in the intervention group who had used the app, and the 95 participants in the control group. Leaving out participants with insufficient accelerometer wear time, data of 220 participants at baseline (134 intervention and 86 control group) and 180 at posttest (104 intervention and 76 control group) were included in the analyses for physical activity and sedentary time; and data of 187 participants at baseline (109 intervention and 78 control group), and 171 at posttest (98 intervention and 73 control group) were included in the analyses for sleep time. Average minutes of non-wear time of the accelerometer at posttest was significantly higher in the intervention group (mean 228, SD 335) compared to the control group (mean 108, SD 256, *P* = 0.005), but not at baseline (intervention: mean 141, SD 266; control: mean 98, SD 202, *P* = 0.70).

Descriptives and group differences at baseline are presented in Table 3. Groups did not differ in terms of gender, grade or family affluence, but the intervention group comprised significantly more participants from the general and technical than from the vocational track. The intervention group spent significantly fewer minutes in light intensity physical activity than the control group, but groups did not differ on the other lifestyle variables or on any of the mental health variables. On average, participants were highly sedentary (9h32min per day), spent 3 h 50 min per day on light intensity physical activity, and 2 h 2 min on moderate to vigorous intensity physical activity. On average, participants slept 8 h 18 min, and the average sleep time difference between week and weekend days was 1 h 16 min. Half of the participants (47.84%) reported taking breakfast every day of the week, whereas 4.67% reported never taking breakfast.

**Table 3:** Baseline demographics and health-related characteristics for each group

	Total (*N* = 279)	Control group (*n* = 95)	Intervention group^a^ (*n* = 150)	Group diff^b^
	***n*** **(%)**	***n*** **(%)**	***n*** **(%)**	***P***
Gender				0.14
Girl	153 (54.84%)	59 (62.11%)	76 (50.67%)	
Boy	124 (44.44%)	36 (37.90%)	72 (48.00%)	
Other	2 (0.72%)	0 (0.00%)	2 (1.33%)	
Education type				< 0.001
General & tech. track	182 (65.23%)	43 (45.26%)	113 (75.33%)	
Vocational track	97 (34.77%)	52 (54.74%)	37 (24.67%)	
Grade				0.15
1^st^ year of secondary	87 (31.18%)	22 (23.16%)	49 (32.67%)	
2^nd^ year of secondary	92 (32.97%)	32 (33.68%)	53 (35.33%)	
3^rd^ year of secondary	100 (35.84%)	41 (43.16%)	48 (32.00%)	
Family Affluence				0.98
Low	80 (28.67%)	28 (29.47%)	45 (30.00%)	
Middle	134 (48.03%)	45 (47.37%)	72 (48.00%)	
High	65 (23.30%)	22 (23.16%)	33 (22.00%)	
	**mean (SD)**	**mean (SD)**	**mean (SD)**	***P***
Mental health				
HRQoL (t-score)	54.11 (6.96)	53.44 (6.40)	54.54 (7.28)	0.12
Psychol. well-being (t-score)	52.10 (8.00)	52.06 (7.34)	52.12 (8.42)	0.70
Moods (t-score)	48.57 (9.77)	48.32 (9.00)	48.73 (10.28)	0.50
Self-Perception (t-score)	52.06 (8.71)	51.97 (8.32)	52.11 (8.97)	0.74
Peer support (t-score)	54.71 (5.97)	54.18 (6.74)	55.04 (5.42)	0.50
Resilience (range 1–5)	3.18 (0.64)	3.16 (0.65)	3.20 (0.64)	0.28
Depressed feelings (t-score)	53.58 (8.53)	54.2 (7.84)	53.4 (9.12)	0.34
Lifestyle behaviors				
LPA^c^ (min/day)	230 (105)	252 (107)	215 (99)	0.001
MVPA^c^ (min/day)	122 (49)	117 (35)	122 (52)	0.89
Sedentary time^c^ (min/day)	572 (127)	587 (116)	570 (132)	0.55
Sleep weekdays^d^ (min/night)	476 (60)	472 (53)	480 (48)	0.14
Sleep weekend^e^ (min/night)	552 (79)	548 (75)	552 (68)	0.74
Breakfast freq. (days/week)	5.15 (2.26)	4.87 (2.38)	5.29 (2.19)	0.30
Sleep quality (range 1–5)	2.87 (0.84)	2.84 (0.83)	2.89 (0.83)	0.63

*Abbreviations*: *LPA* light intensity physical activity, *MVPA* moderate to vigorous intensity physical activity

^a^ Selection of intervention group that used the #LIFEGOALS app (i.e., with in-app duration ≥ 2 min)

^b^ Difference between control and intervention group: χ^2^-test for categorical variables, t-test or Wilcoxon rank test for continuous variables

^c^ Total: *n* = 246, control: *n* = 86, intervention: *n* = 134

^d^ Total: *n* = 221, control: *n* = 88, intervention: *n* = 112

^e^ Total: *n* = 229, control: *n* = 80, intervention: *n* = 125

### Intervention effects

**Fig. 3:**
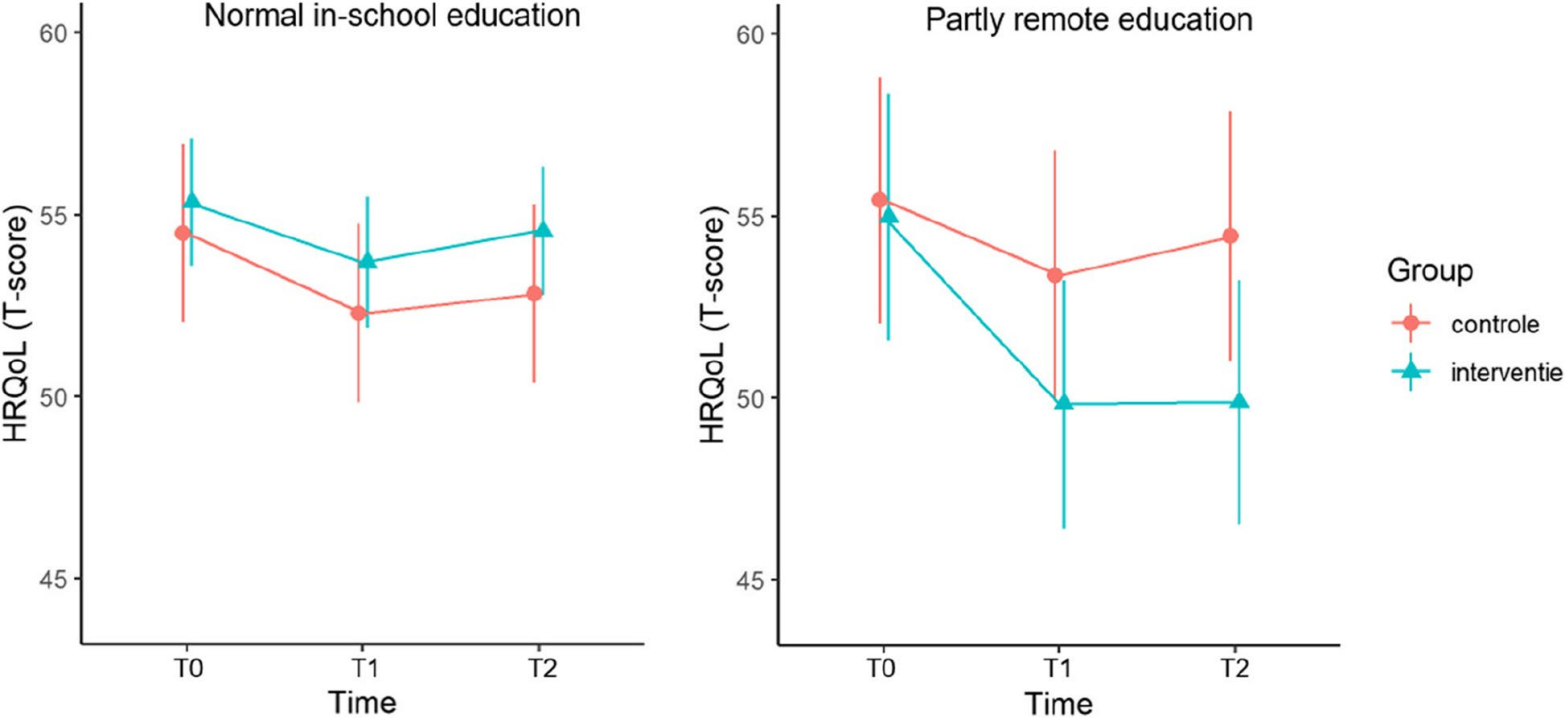
Intervention effects on health-related quality of life. Adjusted means and confidence intervals of health-related quality of life (HRQoL) at baseline (T0), intermittent assessment (T1) and post-intervention (T2) for the subgroup of participants with normal in-school education and the subgroup with partly remote education. The estimates are based on multilevel generalized linear models controlling for age, gender and family affluence

Pandemic-related education restrictions moderated the intervention effect on the primary outcome, HRQoL (*χ*^2^_2_ = 13.13, *P* = 0.001). Among participants who had no pandemic-related education restrictions, there were no effects on HRQoL over time (*χ*^2^_2_ = 0.97, *P* = 0.61); whereas for participants with partly remote education, HRQoL decreased more over time in the intervention than in the control group (*χ*^2^_2_ = 14.72, *P* < 0.001) (see Fig. 3). For psychological well-being, pandemic-related education restrictions did not moderate the intervention effect (*χ*^2^_1_ = 2.20, *P* = 0.14) and there was no significant effect of the intervention (*χ*^2^_1_ = 0.13, *P* = 0.72). Pandemic-related education restrictions moderated the intervention effect on moods (*χ*^2^_1_ = 9.37, *P* = 0.002), self-perception (*χ*^2^_1_ = 4.45, *P* = 0.03) and peer support (*χ*^2^_1_ = 11.10, *P* < 0.001). In case of normal in-school education (i.e., no education restrictions), positive moods increased in the intervention group compared to no change in the control group (*χ*^2^_1_ = 4.14, *P* = 0.04), but no group difference was found for change in peer support (*χ*^2^_1_ = 0.06, *P* = 0.81) or self-perception (*χ*^2^_1_ = 3.22, *P* = 0.07). In case of partly remote education (i.e., education restrictions), positive moods (*χ*^2^_1_ = 6.03, *P* = 0.01) and peer support (*χ*^2^_1_ = 13.69, *P* < 0.001) decreased in the intervention compared to no change in the control group, whereas no group difference was found for self-perception (*χ*^2^_1_ = 1.52, *P* = 0.22). Pandemic-related education restrictions did not moderate the intervention effect on resilience (*χ*^2^_1_ = 0.42, *P* = 0.52) or on depressed feelings (*χ*^2^_1_ = 0.53, *P* = 0.47). There was no significant effect of the intervention on resilience (*χ*^2^_1_ = 0.96, *P* = 0.33) or depressed feelings (*χ*^2^_1_ = 0.45, *P* = 0.50).

Regarding the lifestyle behaviors, pandemic-related sports restrictions but not education restrictions moderated the intervention effect on physical activity (sports: *χ*^2^_1_ = 5.87, *P* = 0.02; education: *χ*^2^_1_ = 2.67, *P* = 0.10) and on sedentary time (sports: *χ*^2^_1_ = 11.96, *P* < 0.001; education: *χ*^2^_1_ = 2.98, *P* = 0.08). For participants who had normal sports possibilities, physical activity decreased significantly less (*χ*^2^_1_ = 4.36, *P* = 0.04) and sedentary time increased significantly less (*χ*^2^_1_ = 6.44, *P* = 0.01) in the intervention compared to the control group. For participants with sports restrictions, there were no intervention effects on physical activity (*χ*^2^_1_ = 1.04, *P* = 0.31) or sedentary time (*χ*^2^_1_ = 0.65, *P* = 0.42). There was no moderation of education restrictions for sleep routine (*χ*^2^_1_ = 0.14, *P* = 0.70), sleep quality (*χ*^2^_1_ = 0.18, *P* = 0.67), or frequency of breakfast consumption (*χ*^2^_1_0.43, *P* = 0.51). Sleep quality significantly increased in the intervention compared to no change in the control group (*χ*^2^_1_ = 6.11, *P* = 0.01). There was no intervention effect on sleep routine (*χ*^2^_1_ = 1.21, *P* = 0.27) or frequency of breakfast consumption (*χ*^2^_1_ = 0.75, *P* = 0.39).

Parameter estimates of the moderation analyses are attached as additional material (Additional file 2). Parameter estimates of the intervention effects are presented in Table 4 and in line graphs (see Fig. 3 and Additional file 3).

**Table 4:** Intervention effects, separate for subgroups in case of significant moderation^a^

Outcome	Subgroup^b^	*β* [95% CI]	*t* test (*df*)	*P*	n
HRQoL T1 vs. T0	In-school	0.55[-1.22; 2.32]	0.61 (419)	0.54	155
	Remote	-3.09[-5.35; -0.82]	-2.67 (232)	0.007	87
HRQoL T2 vs. T0	In-school	0.87[-0.88; 2.63]	0.97 (419)	0.33	155
	Remote	-4.10[-6.28; -1.93]	-3.71 (232)	< 0.001	87
Psych. well-being	Entire sample	0.28[-1.25; 1.82]	0.36 (460)	0.72	242
Moods	In-school	2.76[0.10; 5.43]	2.03 (290)	0.04	155
	Remote	-3.43[-6.17; -0.69]	-2.46 (161)	0.01	87
Self-Perception	In-school	1.62[-0.15; 3.39]	1.79 (290)	0.07	155
	Remote	-1.58[-4.08; 0.93]	-1.23 (161)	0.22	87
Peer support	In-school	0.25[-1.80; 2.29]	0.24 (291)	0.81	155
	Remote	-5.69[-8.71; -2.68]	-3.70 (161)	< 0.001	87
Resilience	Entire sample	0.07[-0.07; 0.22]	0.98 (460)	0.33	242
Depressed feelings	Entire sample	0.58[-1.11; 2.26]	0.67 (459)	0.50	242
PA (ENMO)	Normal	19.11 [1.17; 37.06]	2.09 (100)	0.04	61
	Restricted	3.33 [-3.07; 9.73]	1.02 (279)	0.31	169
SB (min/day)	Normal	-157.98[-279.98; -35.99]	-2.54 (62.43^c^)	0.01	61
	Restricted	19.11 [-27.30; 65.53]	0.81 (136.88^c^)	0.42	169
Sleep routine	Entire sample	13.12 [-10.28; 36.53]	1.10 (168.33^c^)	0.27	210
Sleep quality	Entire sample	0.24 [0.05; 0.43]	2.47 (459)	0.01	242
Breakfast freq	Entire sample	-0.23 [-0.74; 0.28]	-0.87 (229.5^c^)	0.39	242

*Abbreviations*: *PA* physical activity, *ENMO* Euclidean norm minus one, *SB* sedentary behavior

^a^Multilevel generalized linear models testing the interaction effect between Time (T2 vs. T0, unless otherwise specified) and Group (control vs. intervention) controlling for age, gender and family affluence.

^b^In case of significant moderation by pandemic-related education measures, effects are presented for the subgroups ‘normal in-school education’ (In-school) or ‘partly remote education’ (Remote). In case of significant moderation by pandemic-related measures for sports, effects are presented for the subgroups ‘normal sports possibilities’ (Normal) or ‘restricted sports possibilities’ (Restricted). In case of no significant moderation, effects are presented for the entire sample.

^c^Because here a Gauss model was fitted, Satterthwaite’s approximation is used for the calculation of the degrees of freedom.

### Additional analyses

Pandemic-related restrictions for education or sports were dependent on age and grade, and differed depending on the time of data collection (see Additional file 1). Exploration in our sample (see Additional file 4) revealed that all participants with education restrictions were in the 3^rd^ year of secondary education (*χ*^2^_2_ = 245, *p* < 0.001), and participants in the 1^st^ year of secondary education had fewer sports restrictions than participants from the 2^nd^ or 3^rd^ year (*χ*^2^_2_ = 136.14, *p* < 0.001). The groups with or without restrictions did not differ in terms of gender, education type or family affluence.

### Process evaluation interviews

#### Participant characteristics

Due to low response to the invitation messages (10/40, 25%) or low willingness to participate (15/40, 38%), eventually all eligible participants (*n* = 40) were invited for a process evaluation interview. Of the 15 interviewed participants, 2 did not provide the necessary consent forms to include their data. The characteristics of the remaining 13 interviewees are presented in Table 5. Participants had a mean age of 13.45 years (SD 0.92), 54% were girls, and slightly more participants followed the general track (69%) and were in their first year of secondary education (46%). The interviewees had used the app for an average of 1 h over the 3-month intervention period (mean 57, SD 33, Mdn 54, MAD 43 min).

**Table 5:** Characteristics of interviewees (*n* = 13)

	n	mean (SD) app usage in minutes
**Gender**		
Girl	7	68 (32)
Boy	6	45 (31)
**Education type**		
General & technical track	9	62 (31)
Vocational track	4	47 (39)
**Grade**		
1^st^ year of secondary	6	53 (38)
2^nd^ year secondary	3	40 (25)
3^rd^ year of secondary	4	77 (23)
**Family Affluence**		
Low	2	29 (8)
Middle	7	63 (36)
High	4	62 (31)

#### Perceived behavior change processes

All interviewees reported that the intervention had helped them to increase physical activity, improve sleep, reduce sedentary time, and/or increase frequency of breakfast consumption. Upon analysis of interview transcripts, eight main themes were identified: reward system, action and coping planning, self-monitoring, support chatbot, narrative videos, importance of healthy lifestyle, autonomy, social influence and sharing, and physical and situational context. A summary of the findings is presented below. The themes and their subthemes are described in more detail in Additional file 5.

The reward system was experienced as highly motivating to use the app. Users liked that they could earn coins to change the appearance of their avatar, and the challenges in the app were an extra motivator for users to set and reach goals. Users indicated that the action planning, and to a lesser extent also the coping planning, had helped them to improve their health behaviors. The agenda and reminders were experienced as helpful to remember their action plans and to encourage them to put these into practice. Users moreover enjoyed self-monitoring their behavior as it helped them gain insight in their own behavior. This functioned as a trigger to change their behavior, and the monitoring of reached behavioral goals even seemed to help in maintaining behavior change.

The chatbot was experienced as a fun element and was appealing to ask questions to. Participants mainly used the chatbot to better understand the functioning of the app, but also for questions concerning a healthy lifestyle. In case the chatbot could reply to their question, the answers were perceived as useful and facilitated behavior change. However, the chatbot often failed to reply to their specific question, which demotivated further usage of this component. Notifications by the chatbot triggered users to engage with the app and were experienced as a good reminder to set goals.

The narrative videos were rarely watched. About half of the interviewees (6/13) had not initiated any episode. Reasons were that they already had other series to watch, a lack of personal interest in the episodes, forgetfulness, or a lack of time. Interviewees who had watched episodes, were very positive about them and reported that the narrative videos had encouraged usage of the app: the weekly appearance of a new video functioned as an incentive to use the app, and the storyline made viewers realize they should consider changing their behavior towards healthier alternatives.

The intervention as a whole and the usage experience raised awareness about the importance of a healthy lifestyle by aiding in the understanding of the link between behavior and health-related outcomes. Both guidance and autonomy seemed to be important: the choice options and tips for creating action plans raised knowledge and skills on which action plans to set, while the freedom to choose and add own action plans, and to choose when and how to use the intervention, were perceived to have encouraged usage.

Comparing progress and discussing app content with friends was a strong motivator to use the intervention and helped clarify the functioning of the app. If the opinion and usage of classmates would influence user behavior seemed to be context- and person dependent. Adults appeared to exert only a limited impact on adolescents’ user behavior. Specific situations (e.g., boredom) or environment (e.g., sports facilities) encouraged usage of the intervention, and in times of pandemic-related sports restrictions, the app offered alternative options to be active. The opportunity to move research forward was for some adolescents or their parents an additional motivator (to encourage their child) to use the app.

## Discussion

### Summary of the results

This study investigated the effects of the mobile health promotion intervention #LIFEGOALS on adolescents’ mental health and lifestyle behaviors. We found positive intervention effects on physical activity, sedentary behavior, sleep quality, and moods. Pandemic-related measures moderated some of the effects, with beneficial effects on mental health outcomes only present for adolescents who could continue in-school education, and some negative mental health effects for adolescents with partly remote-education. Despite our efforts to enhance engagement, usage attrition was high. Users of the intervention experienced that the reward system and self-regulation techniques had contributed to their behavior change, but also mentioned social factors, quality of technology, autonomy, and the role of gaining awareness and insight in their own behavior.

### mHealth effects on mental health and lifestyle behaviors

#LIFEGOALS was developed to help adolescents adopt a healthy lifestyle as a means to promote mental health. Unfortunately, we found limited effects on mental health. It may well be that effects on positive well-being would still take place in the long-term [67].

The intervention did show beneficial effects on physical activity, sedentary behavior, and sleep quality. In a pandemic situation with normal sports possibilities, using the intervention prevented a decline in daily physical activity and prevented an increase in daily sedentary time. Sleep quality improved regardless of pandemic-related restrictions. In literature, the effects of mHealth interventions on activity-behaviors in adolescents are mixed. One mHealth intervention combined with a Fitbit to promote physical activity in adolescents (the RAW-PA), for example, observed no differences in MVPA post-intervention [68]. There were no intervention effects on sleep routine or frequency of breakfast consumption. Previous sleep interventions have also encountered difficulties for regularizing adolescent weekday-weekend sleep timing [69]. mHealth in general seems to be more effective in improving sleep quality than other dimensions of sleep such as sleep duration [70]. It has been suggested that the bioregulatory and environmental factors of sleep (e.g., late circadian rhythm and early school start times) in adolescence might be at the basis for the lack of intervention effects on sleep routine [71]. Regarding breakfast frequency, a meta-analysis showed that of the few breakfast interventions that observed an increase in frequency of breakfast consumption, making environmental changes proved to be a promising strategy [72]. Although adding objects to the environment is a BCT that seems to be positively related to mHealth effectiveness, it is not often used in health behavior change apps [37]. Future interventions addressing sleep and breakfast routine should consider including ways to induce environmental changes (e.g., place the breakfast clearly visible or remove television from bedroom) to maximize health benefits.

### Impact of pandemic measures

COVID-19 pandemic-related measures limiting in-school education, moderated intervention effects on indicators of mental health: beneficial effects of the intervention were observed only in the group that could continue in-school education, and in contrast, using the intervention negatively influenced HRQoL, moods and perceived support for the subgroup of participants who had to change to partially remote-education. It may well be that using the app might have made participants more aware of the lack of social contact with peers, causing the negative impact by the intervention. Peer relationships and interactions are important in adolescence, and the need to connect with friends from school also plays a role in mHealth [73, 74]. Of note, many interviewed users mentioned social sharing with friends as a motivating factor for using the app. Not being able to readily discuss progress in the app with peers, or the impossibility to plan an activity with a friend when suggested by the app, might have reinforced the negative influence of the pandemic on adolescents’ sense of belonging [75]. Moreover, going to school brings routine into adolescents’ lives and may help them cope with difficulties, especially those who are already vulnerable for mental health issues [76]. Finally, periods without school are also associated with unhealthier lifestyles as regards physical activity, screen time, sleep timing and diet quantity [77, 78]. The realization of this decline (e.g., through self-monitoring in the app) may have negatively impacted mental health.

Pandemic-related measures limiting the sports activities that participants could do, moderated the intervention effects on physical activity and sedentary behavior: only for the group for whom there were almost no restrictions for doing sports, positive effects were found. This might be because the intervention did not anticipate how to promote physical activity or reduce sedentary behavior in a context where organized sports or social activities were greatly limited. Research on the impact of the COVID-19 pandemic confirms that social restrictions and ‘shelter-at-home’ recommendations have made it difficult for adolescents to engage in sports or other forms of organized physical activity, and have caused an increase in leisure screen time and thus sedentary behavior [79]. The #LIFEGOALS intervention was able to counteract these negative trends in case of normal sports possibilities, but not in a context of sports restrictions. Regarding sedentary behavior, especially the parents play an important role in providing boundaries with screen time [79], and a health app might not have been convincing enough to motivate adolescents away from the screen in times when sedentary behavior could not be replaced by sports activities.

Overall, our results point to the influence of context dependency on intervention outcomes. Interventions not only work by its presumed mechanisms of action, but may also require supportive factors (e.g., school and social environment), or may not work in case of contextual hindrances (e.g., COVID-19 measures). There is a need to be transparent and explicit about these factors, which are often overlooked [80]. Identified causal processes can then inform tailoring of mHealth interventions to the context and population [81]. If #LIFEGOALS would have included information and tips on how to be more physically active or how to reduce sedentary behavior in times of the pandemic (i.e., anticipating social and environmental hindrances), this might have had more beneficial effects on lifestyle and mental health.

### User engagement

We attempted to enhance engagement and behavior change by including a chatbot and narrative videos. The chatbot was meant to be an attractive, interactive feature for providing information, for offering support, and for encouraging users to set health action plans. With the narrative, we aimed to reach adolescents with low motivation for health behavior change via entertainment communication, and to pull them back to the app with new episodes of the series. Despite these efforts, attrition rates and sustained usage were similar to interventions without these engagement-enhancing features [82–85].

A first consideration regarding the low engagement is that the chatbot in #LIFEGOALS was not (yet) able to meet user expectations. Users were initially enthusiastic about the chatbot, but felt discouraged to continue using the chatbot when it failed to provide a meaningful reply to their messages [86]. On the other hand, the tailored motivating messages by the chatbot encouraged users to set health behavior goals, pointing to a potentially engagement-enhancing effect. Increasingly, studies are investigating the potential of a support chatbot within mHealth and point to its assets for timely, personalized and cost-effective support [36, 87, 88]. Important concerns, however, include the limitations of the algorithm to provide accurate responses, issues regarding ethics, privacy and security, and technological failures [88, 89]. More studies are needed to invest in the development of a more extensive database and more sophisticated and versatile chatbot to further investigate the potential of an mHealth chatbot for increasing engagement and behavior change.

A second reason for the low engagement may be that adolescents typically show a low interest in health behavior [90]. To grab their attention, an option could be to design mHealth interventions more as entertainment, thereby shifting the focus away from the health concept [91]. The risk of an entertainment-oriented mHealth app, however, is that it must compete with other mobile entertainment available for adolescents. This might be the case with #LIFEGOALS, as only a small number of participants actually watched the narrative. Despite our efforts, our episodes most probably lacked the level of appeal necessary for adolescents to prefer these videos over the extensive offer of series and videos already available via YouTube, Netflix and other channels. Many interviewed users proposed that if friends or influencers would have promoted the app by showing that they used it or by talking positively about it, this would have encouraged them to use the intervention more. Indeed, applying principles from social marketing or the use of social media influencers and memes to mHealth implementation, can influence the popularity, support and reach of mHealth [92, 93]. For adolescents who lack the motivation to change their behavior, it might additionally be necessary to first form health behavior intentions by, for example, raising awareness and improving their health literacy. This may be more easily obtained via other approaches than mHealth (e.g., sensitization activities in school). But also for motivated adolescents, an unstructured stand-alone app might be challenging to engage with due to a lack of integration in their daily life and the absence of fixed moments to work with the app. Alternatives to provide universal health promotion to this age group might be to embed health promotion apps in multiple domains of adolescents’ surroundings and support systems (i.e., community, peers, school, family), for example into a broader health promotion strategy, or to consider non-digital approaches such as nudging or environmental changes [26, 94, 95].

### Process of behavior change

Within mHealth research, it remains an important challenge to identify the intervention components and mechanisms of action that lead to successful behavior change, often referred to as ‘the black box of mHealth’ [96, 97]. Although the current study was not designed to empirically investigate the working mechanisms (no different-component(s) factorial design was run or no mediation analyses were performed [98]), we performed process evaluation interviews with participants who had actively used the app to provide complementary insights. All interviewees reported that #LIFEGOALS had helped them to improve their lifestyle. In terms of BCTs, our findings confirm conclusions from previous research in adolescents pointing to the importance of gamification, self-regulation and reminders. Users perceived that mainly the reward system and the self-regulation techniques (goal setting, action planning, overview in agenda and self-monitoring) had contributed to their behavior change. The prospect to earn coins by setting and completing action plans for personalizing an avatar, is a sort of gamification highly attractive to adolescents [38]. The guidance by the app for creating action and coping plans, whilst also leaving room for autonomy, appears to be an effective mHealth strategy for obtaining behavior change and mental well-being, as do the overview in the agenda, self-monitoring and reminders for planned actions [99–101]. Also social comparison and feedback on behavior by discussing progress and content of the mHealth app with friends, is for some adolescents encouraging to use the intervention [102].

An mHealth intervention is more than a simple combination of BCTs. BCTs should be considered within their context [83, 103], and also other mHealth intervention features determine its success. Jeminiwa and colleagues [38] performed a systematic review upon which the authors developed a theoretical framework outlining five dimensions that should be met concerning mHealth apps for adolescent users. Besides ‘technical quality’, ‘engagement’ (e.g., entertainment, interactivity, gamification) and ‘support system’ (e.g., behavior change support, social support) that are discussed above, they also point to the importance of ‘autonomy’. Autonomy in mHealth can be defined as app accessibility in terms of costs and availability, and as the degree of adolescent control [38]. Users of #LIFEGOALS appreciated that they were given the choice of which behavior(s) they wanted to target and the option to create their own personal action plans. Autonomy is an important motivational force in early adolescence [73], and our findings confirm that this should be taken into account when planning lifestyle interventions for this age group. However, persuasion techniques might be necessary to overcome the risk of reduced intervention exposure in case of complete freedom of use.

Furthermore, several users found it motivating that by using the intervention they gained insight in the actual state of their health behavior. A more accurate image of one’s health behavior can contribute to ‘consciousness raising’ and help a person transition from pre-intender towards an intention to improve their behavior [104]. Moreover, by receiving the intervention, users better understood the link between health behaviors and psychological and social wellbeing, and realized the importance of a healthy lifestyle. The awareness of the importance of a healthy lifestyle appears necessary to avoid compensation of physical activity by unhealthy behavior [105]. It is not entirely clear what aspects of the intervention contributed to the improved awareness of lifestyle importance. One study found that adolescents perceived the Fitbit to increase their awareness [106]. The increased awareness may also partly be a consequence of the intervention complying with the fifth dimension as proposed by Jeminiwa and colleagues [38], being ‘safety, privacy and trust’. mHealth coming from a credible source (in case of #LIFEGOALS, a university) contributes to belief in the information quality, in its turn benefitting attitude towards continuous usage intention [107].

### Strengths and limitations

This study has both strengths and limitations. First, the use of a controlled design allowed to determine whether the intervention was effective while minimizing confounding factors and maximizing statistical reliability. However, randomization was not perfect and the control group comprised a higher proportion of participants from the vocational track. Second, the outcomes were assessed almost immediately after the intervention, and it may well be that effects on mental health will only appear in the longer term as a consequence of a maintained healthy lifestyle [67]. Third, qualitative follow-up interviews provided insights into users’ perceptions of behavior change. However, this was a small and selective group (only participants who had substantially used the app), which could give a biased image of the general user experience. Fourth, accelerometers provide more accurate measurement compared to self-reports about activity behavior. A disadvantage of accelerometry, however, is its higher probability for missing data. We had to deal with lost devices, technical failure, or non-adherence to wearing instructions, for which 18.37% daytime and 26.94% nighttime accelerometry data were lost, compared to only 0,61% missing self-report data. Fifth, this study was run during the COVID-19 pandemic. This is (hopefully) an exceptional event, but it is yet unclear to what extent our results generalize to ‘normal’ times.

## Conclusions

The health effects of #LIFEGOALS were mixed and moderated by pandemic measures. Beneficial effects on physical activity, sedentary behavior and moods were only present in a close-to-normal situation. Detrimental effects on mental health were found for adolescents with partially remote education. Sleep quality improved regardless of pandemic measures. The qualitative findings substantiate the importance of gamification, self-regulation, reminders, increased health awareness, quality of technology, autonomy, and social support for optimal mHealth adoption and effectiveness among an adolescent population.

### Supplementary Information

**Additional file 1.** Pandemic measures.

**Additional file 2.** Moderation analyses.

** Additional file 3.** Line plots of the intervention effects.

** Additional file 4.** Demographic characteristics according to pandemic restrictions.

**Additional file 5.** Qualitative results.

## Data Availability

Used study materials (informed consents, interview guide, web-based surveys), intervention content, the source code of the #LIFEGOALS app, a link to download the app, and the data management plan are available in the Open Science Framework (OSF) repository, https://osf.io/ufskv. The R scripts of the statistical analyses and the anonymized data are also stored on OSF and accessible upon request by contacting the corresponding author.

## Abbreviations

BCT: Behavior change technique
RCT: Randomized controlled trial
HRQoL: Health-related quality of life
PA: Physical activity
SB: Sedentary behavior
MVPA: Moderate to vigorous intensity physical activity

## References

[1] Inchley JC, Stevens GWJM, Samdal O, Currie DB. Enhancing understanding of adolescent health and well-being: the health behaviour in school-aged children study. J Adolesc Health. 2020;66(6). 10.1016/J.JADOHEALTH.2020.03.014 10.1016/j.jadohealth.2020.03.014 32446607

[2] Kaess M, Brunner R, Parzer P (2013). Risk-behaviour screening for identifying adolescents with mental health problems in Europe. Eur Child Adolesc Psychiatry.

[3] Armocida B, Monasta L, Sawyer S (2022). Burden of non-communicable diseases among adolescents aged 10–24 years in the EU, 1990–2019: a systematic analysis of the Global Burden of Diseases Study 2019. Lancet Child Adolesc Health.

[4] Institute of Health Metrics and Evaluation. Global Health Data Exchange (GHDx) . 2019. https://vizhub.healthdata.org/gbd-results?params=gbd-api-2019-permalink/961203b76a31f95c83babc8d8b97f439. Accessed 14 Aug 2022.

[5] Kessler RC, Berglund P, Demler O, Jin R, Merikangas KR, Walters EE (2005). Lifetime prevalence and age-of-onset distributions of DSM-IV disorders in the National Comorbidity Survey Replication. Arch Gen Psychiatry.

[6] Sawyer SM, Azzopardi PS, Wickremarathne D, Patton GC (2018). The age of adolescence. Lancet Child Adolesc Health.

[7] Dray J, Bowman J, Campbell E (2017). Systematic Review of Universal Resilience-Focused Interventions Targeting Child and Adolescent Mental Health in the School Setting. J Am Acad Child Adolesc Psychiatry.

[8] Taylor RD, Oberle E, Durlak JA, Weissberg RP (2017). Promoting positive youth development through school-based social and emotional learning interventions: a meta-analysis of follow-up effects. Child Dev.

[9] World Health Organization. Promoting mental health: concepts, emerging evidence, practice: summary report. 2004. ISBN 92 4 159159 5

[10] Dale H, Brassington L, King K (2014). The impact of healthy lifestyle interventions on mental health and wellbeing: a systematic review. Ment Health Rev J.

[11] Ekkekakis P, ed in chief, Cook D, Craft L, et al., eds. Routledge handbook of physical activity and mental health. 1st ed. Oxon: Routledge; 2013. 10.4324/9780203132678

[12] Rodriguez-Ayllon M, Cadenas-Sánchez C, Estévez-López F (2019). Role of physical activity and sedentary behavior in the mental health of preschoolers, children and adolescents: a systematic review and meta-analysis. Sports Med.

[13] Hoare E, Milton K, Foster C, Allender S (2016). The associations between sedentary behaviour and mental health among adolescents: a systematic review. Int J Behav Nutr Phys Act.

[14] Kandola A, Lewis G, Osborn DPJ, Stubbs B, Hayes JF (2020). Depressive symptoms and objectively measured physical activity and sedentary behaviour throughout adolescence: a prospective cohort study. Lancet Psychiatry.

[15] Sampasa-Kanyinga H, Sampasa-Kanyinga H, Colman I (2020). Combinations of physical activity, sedentary time, and sleep duration and their associations with depressive symptoms and other mental health problems in children and adolescents: a systematic review. Int J Behav Nutr Phys Act.

[16] Chaput JP, Gray CE, Poitras VJ (2016). Systematic review of the relationships between sleep duration and health indicators in school-aged children and youth. Appl Physiol Nutr Metab.

[17] O’Sullivan TA, Robinson M, Kendall GE (2008). A good-quality breakfast is associated with better mental health in adolescence. Public Health Nutr.

[18] Jané-Llopis E (2005). Reviews of evidence: From evidence to practice: mental health promotion effectiveness. Promot Educ.

[19] AAP Committee on School Health (2004). School-based mental health services. Pediatrics.

[20] Bond SJ, Parikh N, Majmudar S (2022). A systematic review of the scope of study of mhealth interventions for wellness and related challenges in pediatric and young adult populations. Adolesc Health Med Ther.

[21] Radovic A, Badawy SM (2020). Technology use for adolescent health and wellness. Pediatrics.

[22] Badawy SM, Kuhns LM. Texting and mobile phone app interventions for improving adherence to preventive behavior in adolescents: a systematic review. JMIR Mhealth Uhealth. 2017;5(4). 10.2196/mhealth.6837 10.2196/mhealth.6837 PMC5415660 28428157

[23] Celik R, Toruner EK (2020). The effect of technology-based programmes on changing health behaviours of adolescents: systematic review. Compr Child Adolesc Nurs.

[24] van Gemert-Pijnen L, Kelders SM, Kip H, Sanderman R (Eds.). eHealth research, theory and development: a multidisciplinary approach. 1st ed. London: Routledge; 2018. 10.4324/9781315385907

[25] Sucala M, Ezeanochie NP, Cole-Lewis H, Turgiss J (2020). An iterative, interdisciplinary, collaborative framework for developing and evaluating digital behavior change interventions. Transl Behav Med.

[26] Vandelanotte C, Müller AM, Short CE (2016). Past, present, and future of ehealth and mhealth research to improve physical activity and dietary behaviors. J Nutr Educ Behav.

[27] Glanz K, Bishop DB (2010). The role of behavioral science theory in development and implementation of public health interventions. Annu Rev Public Health.

[28] Hightow-Weidman LB, Horvath KJ, Scott H, Hill-Rorie J, Bauermeister JA. Engaging youth in mHealth: what works and how can we be sure? Mhealth. 2021;7. 10.21037/MHEALTH-20-48 10.21037/mhealth-20-48 PMC8063019 33898592

[29] Oinas-Kukkonen H, Harjumaa M. Persuasive systems design: key issues, process model, and system features. Commun Assoc Inf Syst. 2009;24(28). 10.17705/1CAIS.02428

[30] Petty RE, Cacioppo JT. The elaboration likelihood model of persuasion. In: Communication and Persuasion. New York: Springer; 1986. p. 1–24. 10.1007/978-1-4612-4964-1_1.

[31] Slater MD, Rouner D (2002). Entertainment-education and elaboration likelihood: understanding the processing of narrative persuasion. Commun Theory.

[32] Perski O, Blandford A, West R, Michie S (2017). Conceptualising engagement with digital behaviour change interventions: a systematic review using principles from critical interpretive synthesis. Transl Behav Med.

[33] Perski O, Crane D, Beard E, Brown J. Does the addition of a supportive chatbot promote user engagement with a smoking cessation app? An experimental study. Digit Health. 2019;5. 10.1177/2055207619880676 10.1177/2055207619880676 PMC6775545 31620306

[34] Crutzen R, Peters GJY, Portugal SD, Fisser EM, Grolleman JJ (2011). An artificially intelligent chat agent that answers adolescents’ questions related to sex, drugs, and alcohol: an exploratory study. J Adolesc Health.

[35] Bickmore TW, Schulman D, Sidner C (2013). Automated interventions for multiple health behaviors using conversational agents. Patient Educ Couns.

[36] Gaffney H, Mansell W, Tai S (2019). Conversational agents in the treatment of mental health problems: mixed-method systematic review. JMIR Ment Health.

[37] Milne-Ives M, LamMEng C, de Cock C, van Velthoven MH, Ma EM (2020). Mobile apps for health behavior change in physical activity, diet, drug and alcohol use, and mental health: systematic review. JMIR Mhealth Uhealth.

[38] Jeminiwa RN, Hohmann NS, Fox BI (2019). Developing a theoretical framework for evaluating the quality of mhealth apps for adolescent users: a systematic review. J Pediatr Pharmacol Ther.

[39] Peuters C, Maenhout L, Crombez G, DeSmet A, Cardon G. Effect evaluation of an mHealth intervention targeting health behaviors in early adolescence for promoting mental well-being (#LIFEGOALS): preregistration of a cluster controlled trial. 10.17605/OSF.IO/3Q5PH.

[40] #LIFEGOALS video episodes. https://tinyurl.com/56tkt9s6. Accessed 23 Dec 2023.

[41] Schwarzer R, Modelo E, De Acción P (2016). Health Action Process Approach (HAPA) as a theoretical framework to understand behavior change. Actualidades en Psicología.

[42] Peuters C, Maenhout L, Crombez G, Lauwerier E, Cardon G, DeSmet A. Development of a mobile healthy lifestyle intervention for promoting mental health in adolescence: #LIFEGOALS. 2022. OSF. 10.17605/OSF.IO/PWUEH.

[43] Currie C, Molcho M, Boyce W, Holstein B, Torsheim T, Richter M (2008). Researching health inequalities in adolescents: the development of the Health Behaviour in School-Aged Children (HBSC) Family Affluence Scale. Soc Sci Med.

[44] Inchley J, Currie D, Budisavljevic S, et al., editors. Spotlight on adolescent health and well-being. Findings from the 2017/2018 Health Behaviour in School-aged Children (HBSC) survey in Europe and Canada. International report. Volume 2. Key data. Copenhagen: WHO Regional Office for Europe; 2020. License: CC BY-NC-SA 3.0 IGO. https://apps.who.int/iris/handle/10665/332104.

[45] Erhart M, Ottova V, Gaspar T (2009). Measuring mental health and well-being of school-children in 15 European countries using the KIDSCREEN-10 Index. Int J Public Health.

[46] Ravens-Sieberer U, Auquier P, Erhart M (2007). The KIDSCREEN-27 Quality of life measure for children and adolescents: psychometric results from a cross-cultural survey in 13 European countries. Qual Life Res.

[47] Ravens-Sieberer U, Gosch A, Rajmil L (2008). The KIDSCREEN-52 Quality of life measure for children and adolescents: psychometric results from a coss-cultural survey in 13 European countries. Value Health.

[48] Ravens-Sieberer U, Erhart M, Rajmil L (2010). Reliability, construct and criterion validity of the KIDSCREEN-10 score: a short measure for children and adolescents’ well-being and health-related quality of life. Qual Life Res.

[49] Robitail S, Ravens-Sieberer U, Simeoni MC (2007). Testing the structural and cross-cultural validity of the KIDSCREEN-27 quality of life questionnaire. Qual Life Res.

[50] Ravens-Sieberer U, the European KIDSCREEN Group. The KIDSCREEN Questionnaires - Quality of Life Questionnaires for Children and Adolescents – Handbook. Lengerich: Pabst Science Publishers; 2006.

[51] Cushing CC, Steele RG (2010). A meta-analytic review of eHealth interventions for pediatric health promoting and maintaining behaviors. J Pediatr Psychol.

[52] Soer R, Six Dijkstra MWMC, Bieleman HJ (2019). Measurement properties and implications of the Brief Resilience Scale in healthy workers. J Occup Health.

[53] Irwin DE, Stucky B, Langer MM (2010). An item response analysis of the pediatric PROMIS anxiety and depressive symptoms scales. Qual Life Res.

[54] Amagai S, Pila S, Kaat AJ, Nowinski CJ, Gershon RC (2022). Challenges in participant engagement and retention using mobile health apps: literature review. J Med Internet Res.

[55] Migueles JH, Rowlands AV, Huber F, Sabia S, van Hees VT. GGIR: a research community–driven open source R Package for generating physical activity and sleep outcomes from multi-day raw accelerometer data. J Meas Phys Behav. 2019;2(3):188–196. 10.1123/jmpb.2018-0063

[56] van Hees VT, Gorzelniak L, Dean León EC, et al. Separating movement and gravity components in an acceleration signal and implications for the assessment of human daily physical activity. PLoS One. 2013;8(4):e61691. 10.1371/journal.pone.0061691. 10.1371/journal.pone.0061691 PMC3634007 23626718

[57] Phillips LRS, Parfitt G, Rowlands AV (2013). Calibration of the GENEA accelerometer for assessment of physical activity intensity in children. J Sci Med Sport.

[58] Kim J, Noh J-W, Kim A, Kwon YD (2022). The impact of weekday-to-weekend sleep differences on health outcomes among adolescent students. Children.

[59] Borazio M, Berlin E, Kucukyildiz N, Scholl PM, van Laerhoven K. Towards a benchmark for wearable sleep analysis with inertial wrist-worn sensing units. 2014 IEEE International Conference on Healthcare Informatics. 2014 Sep 15–17; Verona, Italy. Verona:IEEE; 2014:125–134. 10.1109/ICHI.2014.24

[60] Gariepy G, Danna S, Gobiņa I (2020). How are adolescents sleeping? Adolescent sleep patterns and sociodemographic differences in 24 European and north American countries. J Adolesc Health.

[61] Essner B, Noel M, Myrvik M, Palermo T (2015). Examination of the factor structure of the Adolescent Sleep-Wake Scale (ASWS). Behav Sleep Med.

[62] Riiser K, Løndal K, Ommundsen Y, Småstuen MC, Misvær N, Helseth S. The outcomes of a 12-week internet intervention aimed at improving fitness and health-related quality of life in overweight adolescents: The young & active controlled trial. PLoS One. 2014;9(12). 10.1371/JOURNAL.PONE.0114732 10.1371/journal.pone.0114732 PMC4257715 25478791

[63] Haraldstad K, Christophersen KA, Eide H, Nativg GK, Helseth S (2011). Health related quality of life in children and adolescents: Reliability and validity of the Norwegian version of KIDSCREEN-52 questionnaire, a cross sectional study. Int J Nurs Stud.

[64] Braun V, Clarke V (2006). Using thematic analysis in psychology. Qual Res Psychol.

[65] Nowell LS, Norris JM, White DE, Moules NJ. Thematic analysis. Int J Qual Methods. 2017;16(1). 10.1177/1609406917733847

[66] Maenhout L, Peuters C, Cardon G, Crombez G, DeSmet A, Compernolle S (2022). Nonusage attrition of adolescents in an mhealth promotion intervention and the role of socioeconomic status: secondary analysis of a 2-arm cluster-controlled trial. JMIR Mhealth Uhealth.

[67] Skeen S, Laurenzi CA, Gordon SL (2019). Adolescent mental health program components and behavior risk reduction: a meta-analysis. Pediatrics.

[68] Ridgers ND, Timperio A, Ball K (2021). Effect of commercial wearables and digital behaviour change resources on the physical activity of adolescents attending schools in socio-economically disadvantaged areas: the RAW-PA cluster-randomised controlled trial. Int J Behav Nutr Phys Act.

[69] Illingworth G. The challenges of adolescent sleep. Interface Focus. 2020;10. 10.1098/rsfs.2019.0080 10.1098/rsfs.2019.0080 PMC7202393 32382399

[70] Arroyo AC, Zawadzki MJ (2022). The implementation of behavior change techniques in mhealth apps for sleep: systematic review. JMIR Mhealth Uhealth.

[71] Illingworth G, Sharman R, Harvey CJ, Foster RG, Espie CA (2020). The Teensleep study: the effectiveness of a school-based sleep education programme at improving early adolescent sleep. Sleep Med X.

[72] Harris JA, Carins JE, Rundle-Thiele S (2021). A systematic review of interventions to increase breakfast consumption: a socio-cognitive perspective. Public Health Nutr.

[73] Hawks M, Bratton A, Mobley S, Barnes V, Weiss S, Zadinsky J. Early adolescents’ physical activity and nutrition beliefs and behaviours. Int J Qual Stud Health Well-being. 2022;17(1). 10.1080/17482631.2022.2050523 10.1080/17482631.2022.2050523 PMC8928855 35289233

[74] Seiterö A, Thomas K, Löf M, Müssener U (2021). Using mobile phones in health behaviour change - an exploration of perceptions among adolescents in Sweden. Int J Adolesc Youth.

[75] de Figueiredo CS, Sandre PC, Portugal LCL (2021). COVID-19 pandemic impact on children and adolescents’ mental health: Biological, environmental, and social factors. Prog Neuropsychopharmacol Biol Psychiatry.

[76] Lee J (2020). Mental health effects of school closures during COVID-19. Lancet Child Adolesc Health.

[77] Kuhn AP, Kowalski AJ, Wang Y (2021). On the move or barely moving? Age-related changes in physical activity, sedentary, and sleep behaviors by weekday/weekend following pandemic control policies. Int J Environ Res Public Health.

[78] Zosel K, Monroe C, Hunt E, Laflamme C, Brazendale K, Weaver RG (2022). Examining adolescents’ obesogenic behaviors on structured days: a systematic review and meta-analysis. Int J Obes.

[79] Bates LC, Zieff G, Stanford K (2020). COVID-19 impact on behaviors across the 24-hour day in children and adolescents: physical activity, sedentary behavior, and sleep. Children.

[80] Cartwright. Rigour versus the need for evidential diversity. Synthese. 2021;199:13095–13119. 10.1007/s11229-021-03368-1 10.1007/s11229-021-03368-1 PMC8727433 35058662

[81] Mennis J, McKeon TP, Coatsworth JD, Russell MA, Coffman DL, Mason MJ (2022). Neighborhood disadvantage moderates the effect of a mobile health intervention on adolescent depression. Health Place.

[82] Linardon J, Fuller-Tyszkiewicz M (2020). Attrition and adherence in smartphone-delivered interventions for mental health problems: a systematic and meta-analytic review. J Consult Clin Psychol.

[83] Meyerowitz-Katz G, Ravi S, Arnolda L, Feng X, Maberly G, Astell-Burt T. Rates of attrition and dropout in app-based interventions for chronic disease: systematic review and meta-analysis. J Med Internet Res. 2020;22(9):e20283. 10.2196/20283. 10.2196/20283 PMC7556375 32990635

[84] Chew CSE, Davis C, Lim JKE, et al. Use of a mobile lifestyle intervention app as an early intervention for adolescents with obesity: Single-cohort study. J Med Internet Res. 2021;23(9). 10.2196/20520 10.2196/20520 PMC8512185 34581672

[85] Gorny AW, Chian W, Chee D, Falk Müller-Riemenschneider M. Active use and engagement in an mhealth initiative among young men with obesity: mixed methods study. JMIR Form Res. 2022;6(1). 10.2196/33798 10.2196/33798 PMC8826145 35076399

[86] Maenhout L, Peuters C, Cardon G, Compernolle S, Crombez G, DeSmet A (2021). Participatory development and pilot testing of an adolescent health promotion chatbot. Front Public Health.

[87] Müssener U. Digital encounters: human interactions in mHealth behavior change interventions. Digit Health. 2021;7. 10.1177/20552076211029776 10.1177/20552076211029776 PMC8252401 34262783

[88] Chowdhury MNUR, Haque A, Soliman H. Chatbots: a game changer in mhealth. In: Sixth International Symposium on Computer, Consumer and Control (IS3C). IEEE; 2023:362–366. 10.1109/IS3C57901.2023.00103

[89] Han R, Todd A, Wardak S, Partridge SR, Raeside R. Feasibility and acceptability of chatbots for nutrition and physical activity health promotion among adolescents: systematic scoping review with adolescent consultation. JMIR Hum Factors. 2023;10. 10.2196/43227 10.2196/43227 PMC10199392 37145858

[90] Hoek W, Marko M, Fogel J (2011). Randomized controlled trial of primary care physician motivational interviewing versus brief advice to engage adolescents with an Internet-based depression prevention intervention: 6-month outcomes and predictors of improvement. Transl Res.

[91] Strömmer S, Shaw S, Jenner S (2021). How do we harness adolescent values in designing health behaviour change interventions? A qualitative study. Br J Health Psychol.

[92] Andreasen AR (2004). A social marketing approach to changing mental health practices directed at youth and adolescents. Health Mark Q.

[93] Kostygina G, Tran H, Binns S, et al. Boosting health campaign reach and engagement through use of social media influencers and memes. Soc. Media Soc. 2020;6(2). doi:10.1177/2056305120912475

[94] Bjerre N, Lillefjell M, Magnus E, Anthun KS (2021). Effective interventions targeting the mental health of children and young adults: a scoping review. Scand J Public Health.

[95] Wyatt TH, Bayless AK, Krauskopf P, Gaylord N (2021). Using mhealth applications to promote self-managed health behaviors among teens. J Pediatr Nurs.

[96] Danaher BG, Brendryen H, Seeley JR, Tyler MS, Woolley T (2015). From black box to toolbox: outlining device functionality, engagement activities, and the pervasive information architecture of mHealth interventions. Internet Interv.

[97] Brindal E (2016). The POWeR of looking into the black box. Lancet Diabetes Endocrinol.

[98] Schroé H, Van Dyck D, De Paepe A (2020). Which behaviour change techniques are effective to promote physical activity and reduce sedentary behaviour in adults: A factorial randomized trial of an e- and m-health intervention. Int J Behav Nutr Phys Act.

[99] Bakker D, Kazantzis N, Rickwood D, Rickard N (2016). Mental health smartphone apps: review and evidence-based recommendations for future developments. JMIR Ment Health.

[100] Dugas M, Gao G, Agarwal R. Unpacking mHealth interventions: a systematic review of behavior change techniques used in randomized controlled trials assessing mHealth effectiveness. Digit Health. 2020;6. 10.1177/2055207620905411 10.1177/2055207620905411 PMC7036494 32128233

[101] Thornton L, Gardner LA, Osman B (2021). A multiple health behavior change, self-monitoring mobile app for adolescents: development and usability study of the health4life app. JMIR Form Res.

[102] Tong HL, Laranjo L (2018). The use of social features in mobile health interventions to promote physical activity: a systematic review. NPG Digit Med.

[103] Domin A, Spruijt-Metz D, Theisen D, Ouzzahra Y, Vögele C (2021). Smartphone-based interventions for physical activity promotion: scoping review of the evidence over the last 10 years. JMIR Mhealth Uhealth.

[104] Kersten-van Dijk ET, Westerink JHDM, Beute F, IJsselsteijn WA. Personal informatics, self-insight, and behavior change: a critical review of current literature. Hum Comput Interact. 2017;32(5–6):268–296. 10.1080/07370024.2016.1276456

[105] Beck F, Engel FA, Reimers AK (2022). Compensation or displacement of physical activity in children and adolescents: a systematic review of empirical studies. Children.

[106] Koorts H, Salmon J, Timperio A (2020). Translatability of a wearable technology intervention to increase adolescent physical activity: mixed methods implementation evaluation. J Med Internet Res.

[107] Guo X, Chen S, Zhang X, Ju X, Wang X (2020). Exploring patients’ intentions for continuous usage of mhealth services: elaboration-likelihood perspective study. JMIR Mhealth Uhealth.

