# Supplementary material for: A mobile healthy lifestyle intervention to promote mental health in adolescence: a mixed-methods evaluation
Source: BMC Public Health. 2024 Jan 2;24:44. doi: 10.1186/s12889-023-17260-9 (PMC10763383; doi:10.1186/s12889-023-17260-9)
Supplement: Supplementary file 1 — Additional file 1. Pandemic measures. [file 12889_2023_17260_MOESM1_ESM.docx]

## Additional Material 1. Pandemic measures

Different COVID-19 pandemic-related measures were in force during the data collection of the effect trial. Data collection started at three different moments. The first and second batch of data collection each included four schools (both two intervention and two control schools) and started baseline assessment in, respectively, October and November 2020. The third batch included three schools (two intervention and one control school) and started baseline assessment in January 2021.

### Pandemic-related education measures

From the start of the second batch of data collection onwards, a minimum of 50% distance learning was compulsory for adolescents in the third year of secondary education and above (not affecting 1^st^ and 2^nd^ year) (see Table 1). Following this, the participants in the 1^st^ and 2^nd^ year of secondary school were considered to have ‘no education restrictions’; and the participants in the 3^rd^ year of secondary school were coded to have ‘education restrictions’ for all data collection batches.

**Table 1.** Education restrictions

| **Time** | **Batch 1**  (20/10/2020 – 12/02/2021) | **Batch 2**  (17/11/2020 –  12/03/2021) | **Batch 3**  (11/01/2021 –  29/04/2021) |
| --- | --- | --- | --- |
| T0 | No restrictions | 50% remote education for 3^rd^ year of secondary school | 50% remote education for 3^rd^ year of secondary school |
| IP | Weeks 3-12 with 50% remote education for 3^rd^ year of secondary school | 50% remote education for 3^rd^ year of secondary school | 50% remote education for 3^rd^ year of secondary school |
| T2 | 50% remote education for 3^rd^ year of secondary school | 50% remote education for 3^rd^ year of secondary school | No restrictions |

T0 = baseline assessment, IP = intervention period, T2 = post-intervention assessment

### Pandemic-related sports measures

For persons aged 13 years or above (see Table 2), from the week after the start of the first batch of data-collection onwards, sports indoor were only permitted if these were no-contact sports, and a week later all indoor sports were prohibited and only no-contact outdoor sports were allowed with a maximum of four persons and when keeping distance. By the last weeks of intervention of the third batch of data collection, sports outdoors were allowed again with a maximum of ten persons but adolescents could practice only one hobby. Participants ≥13y were considered having ‘sports restrictions’ when indoors sports were not allowed and when there were many limitations for outdoors sports. Therefore only baseline measurement in the first batch was not coded as ‘sports restrictions’.

Adolescents younger than 13 years could mostly practice sports as normal (see Table 3). Therefore, participants <13y were considered having ‘no sports restrictions’ because they were mostly allowed to keep on performing sports as usual.

**Table 2.** Sports restrictions in force for adolescents ≥13 years old

| **Time** | **Batch 1**  (20/10/2020 – 12/02/2021) | **Batch 2**  (17/11/2020 –  12/03/2021) | **Batch 3**  (11/01/2021 –  29/04/2021) |
| --- | --- | --- | --- |
| T0 | Outdoor allowed;  Indoor only no-contact sports | Outdoor only no-contact sports with max 4pp;  Indoor not allowed | Outdoor only no-contact sports with max 4pp;  Indoor not allowed |
| IP | Outdoor only no-contact sports with max 4pp;  Indoor not allowed | Outdoor only no-contact sports with max 4pp;  Indoor not allowed | Weeks 1-8: same above;  Weeks 9-12: Outdoor allowed to max 10pp with advice to restrict to 1 hobby;  Indoor not allowed |
| T2 | Outdoor only no-contact sports with max 4pp;  Indoor not allowed | Outdoor only no-contact sports with max 4pp;  Indoor not allowed | Outdoor allowed to max 10pp with advice to restrict to 1 hobby;  Indoor not allowed |

T0 = baseline assessment, IP = intervention period, T2 = post-intervention assessment

**Table 3.** Sports restrictions in force for adolescents <13 years old

| **Time** | **Batch 1**  (20/10/2020 – 12/02/2021) | **Batch 2**  (17/11/2020 –  12/03/2021) | **Batch 3**  (11/01/2021 –  29/04/2021) |
| --- | --- | --- | --- |
| T0 | Only minor restrictions | Only minor restrictions | Only minor restrictions |
| IP | Only minor restrictions | Only minor restrictions | Weeks 9-12: indoor advice to restrict to 1 hobby |
| T2 | Only minor restrictions | Only minor restrictions | Indoor advice to restrict to 1 hobby |

T0 = baseline assessment, IP = intervention period, T2 = post-intervention assessment
