# Supplementary material for: A mobile healthy lifestyle intervention to promote mental health in adolescence: a mixed-methods evaluation
Source: BMC Public Health. 2024 Jan 2;24:44. doi: 10.1186/s12889-023-17260-9 (PMC10763383; doi:10.1186/s12889-023-17260-9)
Supplement: Supplementary file 2 — Additional file 2. Moderation analyses. [file 12889_2023_17260_MOESM2_ESM.docx]

## Additional file 4. Demographic characteristics according to pandemic restrictions

**Table.** Differences in demographic characteristics between the groups with or without education or sports restrictions (total N=245)

|  | Group with education restrictions | | | Group with sports restrictions | | |
| --- | --- | --- | --- | --- | --- | --- |
|  | n (%) | *χ*^2^ (*df*) | *P* | n (%) | *χ*^2^ (*df*) | *P* |
| Gender |  | 0.05 (2) | .82 |  | 1.11 (2) | .29 |
| Girl | 47 (52.81%) |  |  | 103 (57.22) |  |  |
| Boy | 40 (44.94%) |  |  | 75 (41.67) |  |  |
| Other | 2 (2.25%) |  |  | 2 (1.11) |  |  |
| Education Type |  | 2.59 (1) | .11 |  | 0.88 (1) | .35 |
| General or technical track | 63 (70.79) |  |  | 111 (61.67) |  |  |
| Vocational track | 26 (29.21) |  |  | 69 (38.33) |  |  |
| Grade |  | 245 (2) | <.001 |  | 136.14 (2) | <.001 |
| 1^st^ | 0 (0.00) |  |  | 16 (8.89) |  |  |
| 2^nd^ | 0 (0.00) |  |  | 75 (41.67) |  |  |
| 3^rd^ | 89 (100.00) |  |  | 89 (49.44) |  |  |
| Family Affluence |  | 4.08 (2) | .13 |  | 3.29 (2) | .19 |
| Low | 20 (22.47) |  |  | 49 (25.16) |  |  |
| Medium | 49 (55.06) |  |  | 92 (50.94) |  |  |
| High | 20 (22.47) |  |  | 39 (23.90) |  |  |
