# Supplementary material for: A mobile healthy lifestyle intervention to promote mental health in adolescence: a mixed-methods evaluation
Source: BMC Public Health. 2024 Jan 2;24:44. doi: 10.1186/s12889-023-17260-9 (PMC10763383; doi:10.1186/s12889-023-17260-9)
Supplement: Supplementary file 3 — Additional file 3. Line plots of the intervention effects. [file 12889_2023_17260_MOESM3_ESM.docx]

## Additional file 5. Qualitative results

***Perceived behavior change mechanisms***

Upon analysis of interview transcripts, eight main themes on how #LIFEGOALS led to improvements in health behavior and/or mental health were identified: reward system, action and coping planning, self-monitoring, support chatbot, narrative videos, importance of healthy lifestyle, autonomy, social influence and sharing, and physical and situational context. The themes and their subthemes are summarized in the table below, where they are also linked to mechanisms of action using the Theory and Technique Tool [1]. The themes are described in more detail below.

**Table.** Identified themes and subthemes on the behavior change mechanisms of #LIFEGOALS linked to MoAs.^a^

| **Themes** and *subthemes* | **MoAs^a^** |
| --- | --- |
| **Reward system** |  |
| *Avatar and coins were fun* | 1 |
| *Presented challenges were extra motivator* | 1 |
| **Action and coping planning** |  |
| *Concrete action plans* | 2 |
| *Agenda as overview of planned activities* | 2, 3 |
| *Reminders of planned behavioral goals* | 4, 3 |
| *Coping planning* | 2 |
| **Self-monitoring** |  |
| *Monitoring was fun* | 1 |
| *Behavioral monitoring gave insight in own behavior* | 3, 5, 6, 8, 9 |
| *Behavioral monitoring triggered behavior change* | 3, 4, 10, 11 |
| *Monitoring of reached behavioral goals helped sustain behavior* | 2, 11 |
| **Support chatbot** |  |
| *Chatbot was fun and clarified how to use intervention* | 1 |
| *Answers by chatbot raised practical knowledge* | 5, 6, 12 |
| *Notifications by chatbot were good reminder to set goals* | 4 |
| **Narrative videos** |  |
| *Weekly videos encouraged app engagement* | 1, 4 |
| *Storyline triggered reflection and copying of behavior* | 10, 8, 9, 13, 14 |
| **The intervention as a whole** |  |
| *Insight in own behavior* | 5, 6, 7, 8, 9 |
| *Importance of healthy lifestyle* | 10, 5, 7, 13 |
| **Autonomy-supporting** |  |
| *Availability of choice options and tips for goals* | 2, 5, 11 |
| *Freedom in usage* | 6, 9 |
| *Combination of multiple behaviors* | 5, 6, 11, 12 |
| **Social influence and sharing** |  |
| *Opinion of peers influenced usage* | 7 |
| *Discussing progress and content with friends encouraged usage* | 7, 11 |
| **Physical and situational context** |  |
| *Physical or environmental opportunities* | 9 |
| *Situational need awareness* | 4, 9, 11 |
| *Research setting* | 9 |

^a^Linked Mechanisms of Action (MoAs) are 1: Reinforcement; 2: Behavioral regulation; 3: Memory, attention & decision processes; 4: Behavioral cueing; 5: Knowledge; 6: Beliefs about capabilities; 7:Perceived susceptibility; 8: Intention 9: Motivation; 7: Social influences and Subjective norms; and 9: Environmental context & resources; 10: Goals; 11: Feedback processes; 12: Skill; 13: Attitude towards the behavior; 14: Social learning/imitation.

Reward system

The reward system (i.e., earning coins to unlock attributes to pimp an avatar) was experienced as highly motivating to set and reach goals. It was almost unanimously perceived as *fun*. Only one user (girl, 14y, general education) did not use it, but could not explain why. Users liked that they could earn coins to pimp up their avatar, and many spontaneously named the reward system as the element that motivated them to use the app.

The challenges in the app, with which new coins could be earned, were an extra *motivator* for users to set and reach goals:

*“I mostly focused on the challenges, I liked that you could earn coins with that and that you could then change your avatar*.” (boy, 13y, B-track)

Action and coping planning

Most users indicated that action planning in the app had helped them to behave in healthier ways (e.g., go to bed earlier, have breakfast more often, be more physically active). Scheduling *concrete plans for action*, facilitated by the options in the app, was experienced as useful and contributing to adherence to the planned behavior.

Most of the interviewees stated that the app aided with planning. It was experienced as positive that an overview of the planned actions became visible in *the agenda*, and this furthermore facilitated recollection of what they had planned and when. As a result, users were more likely to put their action plans into concrete action:

*“Because I used the agenda, I better remembered that I had to take breakfast and do sports, and as a result I have also done that more.*” (girl, 14y, general education)

Some users had even used the app’s agenda for other daily activities (e.g., weekly leisure activities such as basketball training, reading a book for school), showing the utility of the planner.

Especially the notifications reminding the users of their planned activities were experienced as functional. *Reminders* worked both as a memory trigger and as encouragement to complete the action plan:

*“The app helped me to better plan when I do sports, it definitely did, since you get a reminder because otherwise you’re like ‘should I go or not’.*” (boy, 13y, A-track)

To a lesser extent, *coping planning* helped in accomplishing behavioral goals. For some interviewees the coping-planning tools helped them to adapt action plans whenever necessary, or provided them with extra ideas on how to prevent forgetting about their plans. One user explained that thinking about possible obstacles to goals reassured her that the reason for failing does not necessarily depend on her, but also on external factors (girl, 12y, A-track).

Self-monitoring

Users *enjoyed monitoring* their behavior. They liked to see their progress and indicated that this gave them more *insight in their own behavior*:

“*I found the self-monitoring useful, because it showed you when you walked or slept less or more. And then you could start thinking like ‘how come?*’” (girl, 13y, A-track)

Self-monitoring moreover *triggered users to change their behavior*. Mainly monitoring of steps by means of the Fitbit motivated users to get more physically active, but also monitoring breakfast or sleep directed towards healthier lifestyle habits.

Self-monitoring of reached behavioral goals even seemed to help in *maintaining behavior change*:

“*By indicating in the app when I took breakfast, and with that earn coins, the app helped me to eat more in the morning. Because now I almost always take breakfast and before I simply did not.*” (girl, 12y, A-track)

Support chatbot

The chatbot was experienced as a *fun element* and was appealing to adolescents to ask questions to. Participants mainly used the chatbot to better *understand the functioning of the app*, but also for *questions concerning a healthy lifestyle*. Interviewees explained that the chatbot provided them with useful tips to help them “get off the couch” (i.e., to reduce sedentary behavior) (boy, 13y, B-track), or on what to eat for breakfast. As such, the chatbot facilitated change towards a healthier lifestyle:

“*I actually used the chatbot quite often. I found it fun and useful. And I asked what I could eat best, what’s healthy and what not, and what I could better eat in the morning or at noon because some food you better didn’t eat in the morning.”* (girl, 12y, A-track)

Notifications by the chatbot *triggered users* to engage with the app and to set goals. A user who did not receive any notifications by the #LIFEGOALS app, opted that notifications could have encouraged her to open the app. The notifications were experienced as an overload by two interviewees, but even these users experienced the chatbot’s messages as a good reminder to set goals. Users however also ran into the chatbot’s limitations in that it often failed to reply to their specific question: “because yeah, it is a computer” (girl, 12y, A-track).

Narrative videos

Only few users had watched the narrative videos. About half of the interviewees (6/13) had not initiated any episode – they indicated that they already have their own series, that they had no personal interest in the episodes, that they had forgotten that there was a series in the app, or said they were too busy with other things. Interviewees that had watched episodes, were very positive about them:

*“That were some great episodes and fun to watch.”* (boy, 14y, vocational education)

Viewers moreover liked that the videos were not available all at once, but that instead *every week a new episode* appeared:

*“I also prefer one fixed day on which the episodes come online, so I can look forward to it and free up time.”* (girl, 15y, general education)

For some, the narrative *encouraged* them to engage with the broader intervention:

“*To me, the episodes were also an incentive to use the app.*” (girl, 15y, general education)

Watching an episode made some viewers *realize they should consider changing their behavior* towards healthier alternatives, which motivated to make an action plan in the app:

“*By watching the episodes I sometimes thought ‘maybe I should get more physically active and stuff’. And then it was also easy to set those missions.”* (boy, 14y, vocational education)

For one girl, the videos triggered reflection on her own actions and instilled *copying of the characters’ behavior*, although this mainly seemed to concern the copying of behavior or wording that could help her stand up for herself.

The intervention as a whole

Having the intervention at their disposal helped adolescents gain *insight in their current lifestyle*, for which they were more likely to change their behavior:

*“Thanks to that app, I did see ‘oh, I really don’t exercise much’ and then I started exercising more I must say.”* (boy, 12y, B-track)

It also seemed to raise awareness about the *importance of a healthy lifestyle* by aiding in the understanding of the link between behavior and health-related outcomes:

*“The app has helped me to daily take breakfast, because sometimes I really didn’t feel like having breakfast, but it seemed to actually be healthier if you eat in the morning.”* (boy, 13y, B-track)

Users for example noticed that they were less tired, had more energy, and could concentrate better by having a good night’s sleep or by having breakfast.

Autonomy-supporting

The *choice options* *for creating action plans and the tips* on how to obtain certain goals (e.g., tips on how to more easily fall asleep) raised knowledge and skills on which concrete behavior plans to set. The availability of choice and the option to add to the list own ideas, encouraged usage of the app:

*“I found it useful with those missions that for certain things you could do an idea yourself and that you could type in something yourself, that you didn’t necessarily had to choose form the list.”* (girl, 12y, A-track)

Having the *freedom to choose when and how often to use* the intervention was perceived positively. Adolescents would not want to be forced to use the intervention, for it would make them reluctant to do so. In addition, users liked the *combination of multiple behaviors* in the intervention, because it gave them the freedom to choose what behavior(s) they wanted to work on and because it made the app more diverse:

*“I also highly enjoyed using it because there were multiple possibilities. It’s not all focused on one goal, you know.”* (girl, 15y, general education)

Social influence and sharing

The *opinion of friends and peers* in some instances influenced user behavior. Seeing peers using #LIFEGOALS was encouraging. However, when peers did not use the intervention, the intervention was more easily forgotten. For some, the opinion of peers who were no personal friends, was not important. Also if the pupil had a cogent own reason to use the health intervention, a lack of interest by peers did not influence the pupil’s usage:

*“But some classmates didn’t wear the Fitbit or did not use the app or did not think it was useful. But that had no influence for me because my brother is a risk patient.”* (boy, 12y, B-track)

Adults appeared to exert only a limited impact on adolescents’ user behavior. In general, adolescents communicated little about the intervention with their family, and believed their parents and teachers were either positive or neutral about the intervention:

*“They thought it useful because my parents also saw that I went running more and that I was more active.”* (boy, 13y, B-track)

*Discussing progress and content with friends* was a strong motivator to use the intervention. Most interviewees indicated that they liked to talk at school with their friends about their progress, or about what they had (not) done (yet) in the app. The coins moreover installed a kind of competition between friends, encouraging each other to use the app. By means of social sharing, pupils could also help each other in case of unclarity:

*“Yeah, we chatted about the app, especially like ‘did you already earn coins?’ and that was actually quite nice. And then I was like ‘I don’t get it’, and then they helped me how to use the app.*” (girl, 12y, A-track)

Other users, however, did not talk about the intervention and did not seem to need this. As regards Fitbit usage, it worked encouraging if a friend or family member also wore a Fitbit and progress could be compared.

Physical and situational context

Using the intervention did not require changing the user’s environment:

*“The intervention did not change a lot in my life in terms of environment*.” (boy, 14y, technical education)

However, some *physical or environmental opportunities* did influence the extent and content in which the intervention was used. Sports facilities in the environment encouraged usage, and in times of pandemic-related sports restrictions, the app offered alternative options to be active:

*“I once used it when I was in a playground and nearby was something where you could run and then I looked in the app if there was something about running, jog laps or something, and then I set up an action plan and completed it.*” (boy, 13y, B-track)

*Situational need awareness* encouraged usage. For example, the realization that one hadn’t been very healthy so far, made one reach for the app:

*“I sometimes had a day where I didn’t have breakfast or didn’t move that much, and then I had looked at the app, like checking the agenda.”* (girl, 14y, general or technical track)

Or in moments of boredom, for example when hanging in the couch, the adolescent would scroll through the app and be motivated to interrupt the sitting.

Lastly, the fact that by using the intervention they could *help research* move forward, was for some adolescents or their parents an additional motivator (to encourage their child) to use the app.

***Reference***

1. Johnston M, Carey RN, Connell Bohlen LE, et al. Development of an online tool for linking behavior change techniques and mechanisms of action based on triangulation of findings from literature synthesis and expert consensus. Translational Behavioral Medicine. 2021;11(5):1049–1065. doi:10.1093/tbm/ibaa050
