# Supplementary material for: A mobile healthy lifestyle intervention to promote mental health in adolescence: a mixed-methods evaluation
Source: BMC Public Health. 2024 Jan 2;24:44. doi: 10.1186/s12889-023-17260-9 (PMC10763383; doi:10.1186/s12889-023-17260-9)
Supplement: Supplementary file 4 — Additional file 4. Demographic characteristics according to pandemic restrictions. [file 12889_2023_17260_MOESM4_ESM.docx]

## **
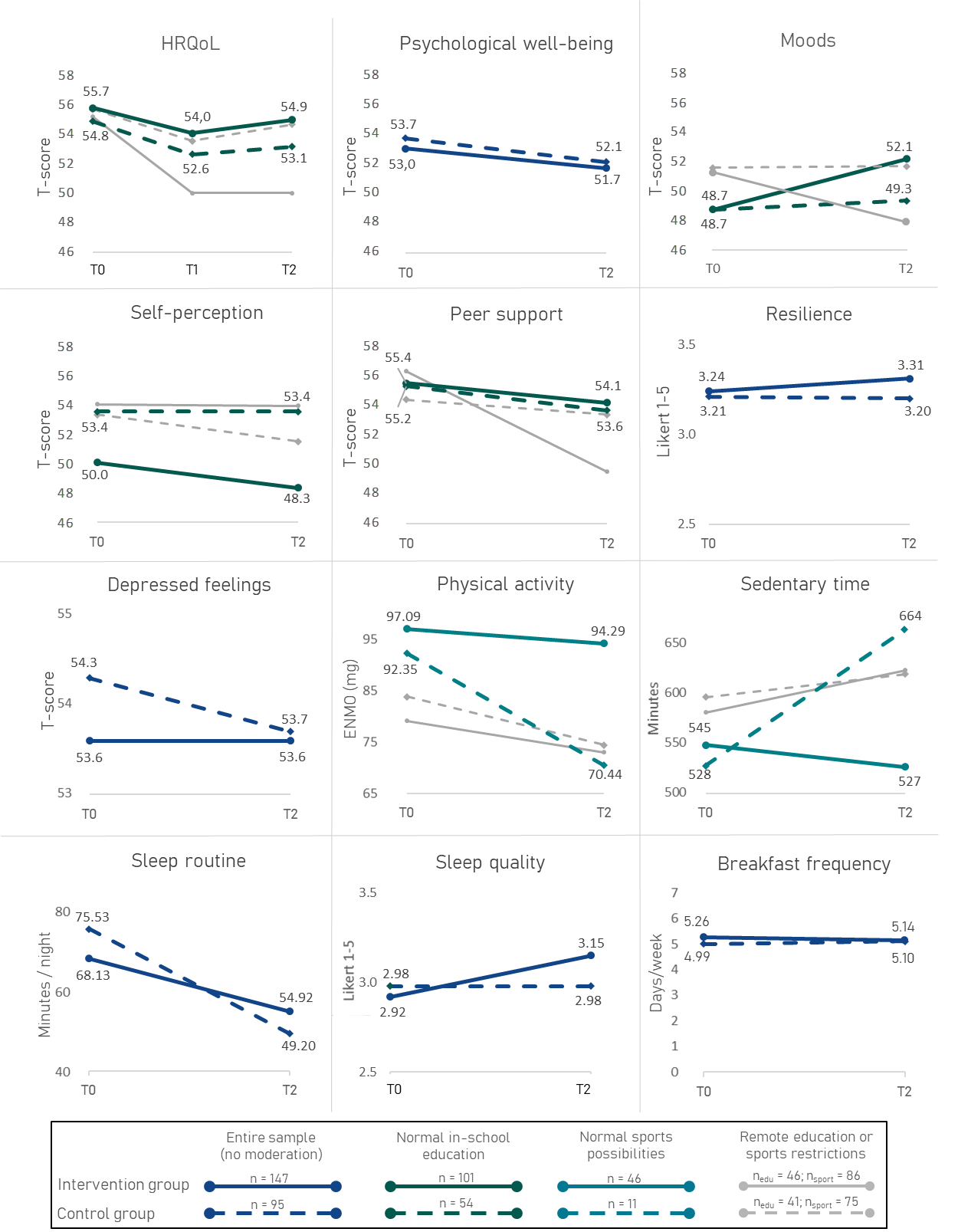
**Additional file 3. Line plots of the intervention effects

**Figure.** Line plots showing the group*time effects on the mental health outcomes and lifestyle behaviors. In case of moderation, adjusted means are presented for the subgroup closest to a normal (non-pandemic-restricted) situation, and the subgroup with pandemic-related restrictions for education/sports is represented in grey. Full lines are the intervention group, dotted lines the control group. The estimates are based on multilevel generalized linear models controlling for age, gender and family affluence.
