# Supplementary material for: A mobile healthy lifestyle intervention to promote mental health in adolescence: a mixed-methods evaluation
Source: BMC Public Health. 2024 Jan 2;24:44. doi: 10.1186/s12889-023-17260-9 (PMC10763383; doi:10.1186/s12889-023-17260-9)
Supplement: Supplementary file 5 — Additional file 5. Qualitative results. [file 12889_2023_17260_MOESM5_ESM.docx]

## Additional file 2. Moderation analyses

**Table.** Estimates of the moderation by pandemic-related education and/or sports restrictions on intervention effects^a-b^

|  | Education restrictions | | | | Sports restrictions | | | |  |
| --- | --- | --- | --- | --- | --- | --- | --- | --- | --- |
|  | *β* [95%CI] | *t* | *df* | *P* | *β* [95%CI] | *t* | *df* | *P* | |
| **Mental health** |  |  |  |  |  |  |  |  | |
| HRQoL T1 vs. T0 | 3.62 [0.75; 6.48] | 2.47 | 1 | .013 |  |  |  |  | |
| HRQoL T2 vs. T0 | 4.99 [2.21; 7.78] | 3.51 | 1 | <.001 |  |  |  |  | |
| Psychological well-being | 2.35 [-0.75; 5.45] | 1.49 | 1 | .14 |  |  |  |  | |
| Moods | 6.26 [2.25; 10.27] | 3.06 | 1 | .002 |  |  |  |  | |
| Self-Perception | 3.20 [0.23; 6.18] | 2.11 | 1 | .035 |  |  |  |  | |
| Peer support | 5.92 [2.44; 9.40] | 3.33 | 1 | <.001 |  |  |  |  | |
| Resilience | 0.10 [-0.20; 0.40] | 0.65 | 1 | .52 |  |  |  |  | |
| Depressed feelings | -1.28 [-4.72; 2.16] | -0.73 | 1 | .47 |  |  |  |  | |
| **Lifestyle behaviors** |  |  |  |  |  |  |  |  | |
| PA (ENMO) | -10.51 [-23.12; 2.09] | -1.64 | 1 | .10 | 20.99 [4.01; 37.67] | 2.42 | 1 | .02 | |
| SB (min/day)^d^ | 87.68 [-11.84; 186.89] | 1.73 | 1 | .09 | -215.84 [-338.16; -93.52] | -3.46 | 1 | <.001 | |
| Sleep routine (min) | -8.84 [-54.55; 36.86] | -0.38 | 1 | .71 |  |  |  |  | |
| Sleep quality | 0.08 [-0.30; 0.47] | 0.43 | 1 | .67 |  |  |  |  | |
| Breakfast (times/week) | 0.36 [-0.70; 1.41] | 0.66 | 1 | .51 |  |  |  |  | |

Abbreviations: PA = physical activity; ENMO = Euclidean Norm Minus One, expressed in milligravity-based acceleration units (*mg*); SB = sedentary behavior

^a^Multilevel generalized linear models testing the interaction effect between Time (T2 vs. T0, unless otherwise specified) and Group (control vs. intervention) in interaction with Education restrictions (remote vs. in-school education) and Sports restrictions (yes/no restrictions), controlling for age, gender and family affluence.

^b^N=242, except for PA and SB (N=230) and sleep time difference (N=210)
